# A multicentre validation study of the diagnostic value of plasma neurofilament light

**DOI:** 10.1038/s41467-021-23620-z

**Published:** 2021-06-07

**Authors:** Nicholas J. Ashton, Shorena Janelidze, Ahmad Al Khleifat, Antoine Leuzy, Emma L. van der Ende, Thomas K. Karikari, Andrea L. Benedet, Tharick A. Pascoal, Alberto Lleó, Lucilla Parnetti, Daniela Galimberti, Laura Bonanni, Andrea Pilotto, Alessandro Padovani, Jan Lycke, Lenka Novakova, Markus Axelsson, Latha Velayudhan, Gil D. Rabinovici, Bruce Miller, Carmine Pariante, Naghmeh Nikkheslat, Susan M. Resnick, Madhav Thambisetty, Michael Schöll, Gorka Fernández-Eulate, Francisco J. Gil-Bea, Adolfo López de Munain, Ammar Al-Chalabi, Pedro Rosa-Neto, Andre Strydom, Per Svenningsson, Erik Stomrud, Alexander Santillo, Dag Aarsland, John C. van Swieten, Sebastian Palmqvist, Henrik Zetterberg, Kaj Blennow, Abdul Hye, Oskar Hansson

**Affiliations:** 1 Department of Psychiatry and Neurochemistry, Institute of Neuroscience & Physiology, The Sahlgrenska Academy at the University of Gothenburg, Mölndal, Sweden; 2 Wallenberg Centre for Molecular and Translational Medicine, University of Gothenburg, Gothenburg, Sweden; 3 Department of Old Age Psychiatry, Maurice Wohl Clinical Neuroscience Institute, King’s College London, London, UK; 4 NIHR Biomedical Research Centre for Mental Health & Biomedical Research Unit for Dementia at South London & Maudsley NHS Foundation, London, UK; 5 Clinical Memory Research Unit, Department of Clinical Sciences Malmö, Lund University, Lund, Sweden; 6 Department of Basic and Clinical Neuroscience, Maurice Wohl Clinical Neuroscience Institute, King’s College London, London, UK; 7 Department of Neurology and Alzheimer Center, Erasmus University Medical Center, Rotterdam, Netherlands; 8 Translational Neuroimaging Laboratory, The McGill University Research Centre for Studies in Aging, Montreal, QC Canada; 9 Montreal Neurological Institute, Montreal, QC Canada; 10 Memory Unit, Department of Neurology, Hospital de la Santa Creu i Sant Pau, Barcelona, Spain; 11 CIBERNED, Centro de Investigación Biomédica en Red de Enfermedades Neurodegenerativas, Instituto de Salud Carlos III, Madrid, Spain; 12 Laboratory of Clinical Neurochemistry, Neurology Clinic, University of Perugia, Perugia, Italy; 13 Fondazione IRCCS Ca’ Granda, Ospedale Policlinico, Milan, Italy; 14 Dino Ferrari Center, University of Milan, Milan, Italy; 15 Department of Neuroscience, Imaging and Clinical Sciences, University G.d’Annunzio of Chieti-Pescara, Chieti, Italy; 16 Neurology Unit, Department of Clinical and Experimental Sciences, University of Brescia, Brescia, Italy; 17 Parkinson’s Disease Rehabilitation Centre, FERB ONLUS, Trescore Balneario, BG Italy; 18 Department of Clinical Neuroscience, Institution of Neuroscience and Physiology, Sahlgrenska Academy, University of Gothenburg, Gothenburg, Sweden; 19 Department of Neurology, Sahlgrenska University Hospital, Gothenburg, Sweden; 20 Department of Health Sciences, University of Leicester, Leicester, UK; 21 Department of Neurology, University of California San Francisco, Memory and Aging Center, San Francisco, CA USA; 22 Department of Radiology & Biomedical Imaging, University of California, San Francisco, CA USA; 23 Stress, Psychiatry and Immunology Lab & Perinatal Psychiatry, Maurice Wohl Clinical Neuroscience Institute, King’s College London, London, UK; 24 Brain Aging and Behavior Section, Laboratory of Behavioral Neuroscience, National Institute on Aging, National Institutes of Health, Baltimore, MD USA; 25 Clinical and Translational Neuroscience Section, Laboratory of Behavioral Neuroscience, National Institute on Aging, National Institutes of Health, Baltimore, MD USA; 26 Department of Neurodegenerative Disease, Queen Square Institute of Neurology, University College London, London, UK; 27 Neurosciences Area, Biodonostia Health Research Institute, San Sebastián, Spain; 28 Department of Neurology, Donostia University Hospital, Osakidetza, San Sebastián, Spain; 29 CIBERNED, Carlos III Health Institute, Madrid, Spain; 30 Department of Neurosciences, Basque Country University, San Sebastián, Spain; 31 Department of Neurology, King’s College Hospital, London, UK; 32 Department of Forensic and Neurodevelopmental Sciences, Institute of Psychiatry, Psychology and Neuroscience, King’s College London, London, UK; 33 South London and Maudsley NHS Foundation Trust, London, UK; 34 London Down Syndrome Consortium (LonDowns), London, UK; 35 Department of Clinical Neuroscience, Karolinska Institutet, Stockholm, Sweden; 36 Memory Clinic, Skåne University Hospital, Malmö, Sweden; 37 Centre for Age-Related Medicine, Stavanger University Hospital, Stavanger, Norway; 38 Department of Neurology, Skåne University Hospital, Lund University, Lund, Sweden; 39 Clinical Neurochemistry Laboratory, Sahlgrenska University Hospital, Mölndal, Sweden; 40 UK Dementia Research Institute, University College London, London, UK

**Keywords:** Neurodegeneration, Diagnostic markers, Predictive markers

## Abstract

Increased cerebrospinal fluid neurofilament light (NfL) is a recognized biomarker for neurodegeneration that can also be assessed in blood. Here, we investigate plasma NfL as a marker of neurodegeneration in 13 neurodegenerative disorders, Down syndrome, depression and cognitively unimpaired controls from two multicenter cohorts: King’s College London (*n* = 805) and the Swedish BioFINDER study (*n* = 1,464). Plasma NfL was significantly increased in all cortical neurodegenerative disorders, amyotrophic lateral sclerosis and atypical parkinsonian disorders. We demonstrate that plasma NfL is clinically useful in identifying atypical parkinsonian disorders in patients with parkinsonism, dementia in individuals with Down syndrome, dementia among psychiatric disorders, and frontotemporal dementia in patients with cognitive impairment. Data-driven cut-offs highlighted the fundamental importance of age-related clinical cut-offs for disorders with a younger age of onset. Finally, plasma NfL performs best when applied to indicate no underlying neurodegeneration, with low false positives, in all age-related cut-offs.

## Abstract (web summary)

Cerebrospinal fluid neurofilament light (NfL) is a biomarker for neurodegeneration that can also be assessed in blood. Here the authors show in a validation study the potential for plasma NfL as a biomarker for several neurodegenerative diseases.

## Introduction

In the management of neurological disorders, reliable and easily accessible biomarkers are needed to recognize or rule out an underlying neurodegenerative process contributing to cognitive decline at the earliest stage. Cerebrospinal fluid (CSF) biomarkers for amyloid-β (Aβ42), total tau (T-tau), and phosphorylated tau (P-tau) work well to identify certain neurodegenerative disorders such as Alzheimer’s disease (AD) and its underlying pathology^1^ and are central to the biological definition of the disease^2^. However, at this time, no such fluid biomarkers are available for other common or rarer neurodegenerative disorders.

Axonal degeneration or injury is a predominant feature of many neurodegenerative disorders that results in irreversible impairment. In response to such damage, neurofilament light chain (NfL), a structural component of the neural cytoskeleton, is released into the extracellular space initiating a concentration increase in the CSF^3^. These elevations are observed in the majority of neurodegenerative disorders^4^ along with inflammatory^5^, traumatic^6^, and vascular conditions^7^. Nonetheless, even under normal circumstances, NfL is continuously released from axons in an age-dependent manner with typical NfL reference ranges in the CSF increasing by twofold between ages 20–50 years and further doubling by the age of 70^8,9^. A considerable drawback of CSF NfL, and consequently all CSF biomarkers, is the perceived invasiveness or complexity attached to lumbar punctures which will undoubtedly limit use for routine clinical assessment.

Recent advances in ultrasensitive immunological assays^10–14^ and immunoprecipitation mass spectrometry (IPMS) methods^15–17^ have been developed for the quantification of Aβ42/Aβ40^12,15,16^ and P-tau^11,13,18,19^ in blood, and like CSF, they demonstrate high specificity for AD-type pathology. NfL can be quantified at femtomolar concentrations in plasma or serum, which has enabled the reliable detection of NfL not only in symptomatic patients but also in cognitively unimpaired (CU) individuals of all ages^20^. A key advantage of peripheral NfL over other postulated blood biomarkers is that it shows a strong correlation to CSF NfL levels across several diagnostic groups, supporting the notion that blood NfL reflects central nervous system pathophysiology with negligible peripheral interference. Consequently, numerous CSF NfL findings have been replicated in blood, including increased concentrations of blood NfL in AD^21–23^, frontotemporal dementia (FTD)^24^, and several other disorders (for review see ref. ^25^). Interestingly, NfL is seemingly not elevated in Parkinson’s disease (PD) in comparison to other neurodegenerative disorders and therefore discrimination can be made from atypical parkinsonian disorders^26,27^. Furthermore, developing evidence demonstrates the potential use of using plasma NfL in discriminating FTD and primary psychiatric disorders^28,29^. This suggests that plasma does have differential diagnostic potential in clinically relevant situations.

The context of the use of a blood biomarker, such as NfL, is in primary care or memory clinics care where it could be used as a rapid screening tool to identify or reject neurodegeneration as an underlying cause of cognitive symptoms^30^. To achieve this at the individual level, reference values to indicate neurodegeneration need to be established which results in a low rate of false positives. In this study, we examined 2269 individuals from two independent multicentre cohorts to first demonstrate the distributions of plasma NfL in CU individuals, the AD continuum and a broad range of neurodegenerative disorders, Down syndrome, and depression. Second, we examined the diagnostic utility of plasma NfL in terms of effect size, the area under the curve (AUC), specificity, and sensitivity when differentiating relevant neurodegenerative diseases from each other and CU individuals. Finally, age-related and data-driven plasma NfL concentration cutoffs were derived to indicate neurodegeneration and these were tested to predict the prevalence of abnormal NfL in neurodegenerative disorders, Down syndrome, depression, and CU individuals.

## Results

The demographic and clinical data for the KCL and Lund cohorts are displayed in Tables 1 and 2. A full description of the demographic variables and the relation of plasma NfL with age, sex, *APOE* ε4 carrier status, disease severity measures, and CSF NfL are fully are described in Supplementary Results 1–3 and presented in Supplementary Results Tables 2–4.

**Table 1:** Demographics of the KCL cohort.

		Cognitively unimpaired		Cognitive impairment						Parkinsonian		Other			
Characteristics		CU Aβ−	CU Aβ+	MCI Aβ−	MCI Aβ+	EOAD	AD	FTD	PDD/DLB	PD	CBS/PSP	DS	DSAD	ALS	Depression
Number	805	130	28	55	31	59	102	54	59	140	19	29	12	50	37
Age, years, mean (SD)	63.6 (14.6)	64.0 (14.1)	66.3 (7.7)	70.7 (6.36)	69.6 (7.0)	57.8 (5.2)	76.1 (6.2)	65.4 (9.5)	77.5 (6.4)	65.5 (10.0)	70.4 (8.4)	50.4 (11.4)	59.1 (9.8)	62.2 (12.5)	32.3 (6.9)
Female, *n* (%)	413 (48.5)	63 (54.8)	13 (46.4)	29 (52.7)	16 (51.6)	32 (49.2)	55 (53.9)	23 (42.6)	32 (54.2)	52 (37.1)	10 (52.6)	10 (34.5)	4 (33.3)	13 (26.0)	35 (68.6)
APOE e4 carriers, *n* (%) [missing]	131 (35.1) [479]	18 (28.1) [51]	8 (30.8) [2]	20 (38.5) [3]	15 (50.0) [1]	8 (34.8) [42]	43 (47.3) [11]	9 (25.0) [18]			3 (25.0) [7]	4 (14.8) [2]	3 (25.0)		
MMSE, mean (SD)	24.3 (5.8)	29.4 (1.0)	29.0 (1.0)	26.0 (3.2)	26.0 (2.2)	22.2 (6.5)	19.9 (6.7)	25.9 (4.5)	20.2 (4.2)	27.3 (2.6)	23.1 (5.3)				
UPDRS-III	24.8 (10.9) [24]								20.1 (2.3)	17.8 (10.3) [22]	36.5 (20.3) [2]				
Hoehn & Yahr scale	2.3 (0.7) [24]								2.2 (0.5)	1.9 (0.6) [22]	2.8 (0.9) [2]				
Diagnostic delay, months [missing]	7.8 (6.9) [7]													7.8 (6.9) [7]	
HAM-D															>13 = 15 >17 = 22
CSF Aβ, mean (SD)	675 (352)	999 (317)	405 (103)	921 (385)	413 (120)		434 (155)								
Aβ PET	1.55 (0.5)	1.29 (0.1)	1.81 (0.3)				2.08 (0.4)	1.22 (0.3)			1.61 (0.2)

**Table 2:** Demographics of the Lund cohort.

		Cognitively unimpaired				Cognitive impairment							Parkinsonian		
Characteristics		CU Aβ−	CU Aβ+	SCD Aβ−	SCD Aβ+	MCI Aβ−	MCI Aβ+	EOAD	AD	FTD	PDD/DLB	VaD	PD	CBS/PSP	MSA
Number	1464	273	103	134	75	115	165	23	134	150	46	22	171	24	29
Age, years, mean (SD)	70 (7.1)	72 (6.5)	73 (5.5)	69 (5.7)	72 (5.3)	69 (5.6)	72 (4.5)	59 (3.5)	75 (5.2)	64 (8.8)	73 (6.9)	64 (5.7)	65 (10.7)	72 (4.4)	76 (4.4)
Female, *n* (%)	734 (50.1)	160 (58.6)	64 (62.1)	77 (57.5)	32 (42.7)	36 (31.3)	79 (47.9)	14 (60.9)	79 (59.0)	77 (51.3)	15 (32.6)	13 (44.8)	63 (36.8)	15 (62.5)	10 (45.5)
APOE e4 carriers, *n* (%) [missing]	507 (40.4) [210]	46 (17.1) [4]	65 (63.1) [0]	33 (24.8) [1]	48 (64.0) [0]	27 (23.5) [0]	116 (70.3) [0]	6 (54.5) [12]	81 (66.4) [12]		18 (41.9) [3]	9 (45.0) [2]	44 (29.9) [24]	7 (35.0) [4]	6 (22.2) [2]
MMSE, mean (SD) [missing]	26.8 (3.8) [96]	29.0 (1.1) [2]	28.8 (1.1) [0]	28.7 (1.3) [4]	28.2 (1.6) [5]	27.3 (1.9) [2]	26.8 (1.8) [4]	20.6 (4.5) [1]	21.3 (4.0) [3]	23.3 (5.8) [51]	22.3 (3.9) [7]	22.3 (4.5) [11]	28.0 (2.0) [0]	25.5 (4.5) [4]	27.2 (2.7) [2]
UPDRS-III [missing]	33.75 (15.1) [49]										34.4 (14.7) [20]		16.9 (10.4) [23]	41.9 (18.5) [4]	41.8 (22.1) [2]
Hoehn & Yahr scale [missing]	3.2 (0.9) [49]										3.0 (0.8) [20]		1.9 (0.8) [23]	3.9 (1.0) [4]	3.9 (1.1) [2]
CSF Aβ42/Aβ40	0.10 (0.04)	0.13 (0.02)	0.06 (0.02)	0.13 (0.02)	0.06 (0.02)	0.13 (0.03)	0.06 (0.02)	0.06 (0.04)	0.07 (0.03)	N/A	0.11 (0.04)	0.13 (0.02)	0.12 (0.03)	0.11 (0.03)	0.08 (0.03)
CSF NfL	1321 (1158)	894 (515)	1107 (770)	935 (470)	1332 (887)	1599 (1495)	1549 (1162)	1066 (870)	1845 (1572)	4590 (1230)	1589 (1030)	1758 (794)	888 (640)	2577 (1345)	3435 (1280)

### Plasma NfL concentrations in cognitively unimpaired and neurodegenerative disorders

**Fig. 1:**
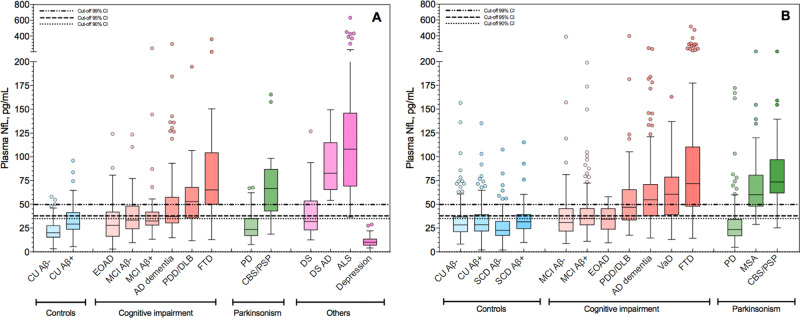
The concentrations of plasma NfL for different diagnostic and controls groups in the KCL and Lund cohorts. Plasma neurofilament light (NfL) in different diagnostic groups; KCL (**A** *n*  =  805) and Lund (**B** *n * =  1464) cohorts. For each plot, the horizontal bar shows the median, and the upper and lower boundaries show the 25th and 75th percentiles, respectively. Source data are provided as a Source Data file. KCL Cohort—AD    Alzheimer’s disease (*n* = 102), ALS   amyotrophic lateral sclerosis (*n* = 50), CU Aβ− cognitively unimpaired without Aβ pathology (*n* = 130), CU Aβ+ cognitively unimpaired with Aβ pathology (*n* = 28), CBS/PSP   corticobasal syndrome and progressive supranuclear palsy (*n* = 19), depression (*n* = 37), DS    Down syndrome (*n* = 29), DSAD   Down syndrome Alzheimer’s disease (*n* = 12), EOAD   early-onset Alzheimer’s disease (*n* = 59), FTD   frontotemporal dementia (*n* = 54), MCI Aβ− mild cognitive impairment without Aβ pathology (*n* = 55), MCI Aβ+ mild cognitive impairment with Aβ pathology (*n* = 31), PD   Parkinson’s disease (*n* = 140), PDD/DLB   Parkinson’s disease dementia and dementia with Lewy bodies (*n* = 59). Lund Cohort—AD   Alzheimer’s disease (*n* = 134), CU Aβ− cognitively unimpaired without Aβ pathology (*n* = 273), CU Aβ+ cognitively unimpaired with Aβ pathology (*n* = 103), CBS/PSP   corticobasal syndrome and progressive supranuclear palsy (*n* = 24), EOAD   early-onset Alzheimer’s disease (*n* = 23), FTD   frontotemporal dementia (*n* = 150), MCI Aβ− mild cognitive impairment without Aβ pathology (*n* = 115), MCI Aβ+ mild cognitive impairment with Aβ pathology (*n* = 165), MSA   multiple system atrophy (*n* = 29), PD    Parkinson’s disease (*n* = 171), PDD/DLB   Parkinson’s disease dementia and dementia with Lewy bodies (*n* = 46), SCD Aβ− subjective cognitive decline without Aβ pathology (*n* = 134), SCD Aβ+ subjective cognitive decline with Aβ pathology (*n* = 75), VaD vascular dementia (*n* = 22).

Plasma NfL levels (unadjusted for age) for CU and diagnostic groups in the KCL and Lund cohorts are displayed in Fig. 1A and B, respectively. In the KCL cohort, the concentrations of plasma NfL were significantly increased in all cognitively impaired, parkinsonian, DSAD, and ALS compared to the CU Aβ− group (*P* < 0.0001, Fig. 1A), with the exception of PD, DS, depression, and EOAD groups. When adjusting for age, EOAD patients had significantly higher NfL levels as compared to those of CU Aβ− group (*P* = 0.001). Highly significant increases of plasma NfL were observed in all cognitively impaired and atypical parkinsonian groups as compared to PD (*P* < 0.0001). However, FTD and ALS were the only groups showing significantly higher plasma NfL levels in comparison to AD dementia (*P* < 0.05 and *P* < 0.0001, respectively). Plasma NfL levels in CU Aβ+ were also significantly higher as compared to CU Aβ− individuals (*P* < 0.05).

Similar findings were found in the Lund cohort where the concentrations of plasma NfL were significantly increased in all disorders when compared to the CU Aβ−, CU Aβ+ , SCD Aβ−, SCD Aβ+ groups (*P* < 0.0001), and MCI groups (*P* = 0.001), with nonsignificant differences in PD and EOAD. Age adjustment did demonstrate a significant increase in the EOAD patients in comparison to Aβ− (*P* = 0.001) but not to Aβ+ control groups. However, unlike the KCL cohort, when comparing unimpaired groups, no significant increase was observed in CU Aβ+, SCD Aβ−, and SCD Aβ+ when compared with CU Aβ− individuals. AD dementia was significantly increased compared with all CU, MCI, and PD groups (*P* < 0.0001), as well as EOAD (*P* < 0.005), but not significantly different from EOAD after age correction.

**Fig. 2:**
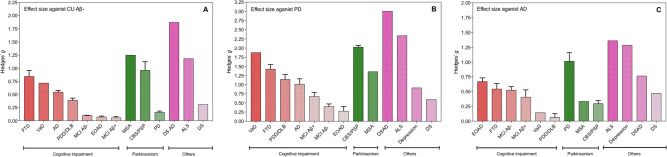
Effect sizes of neurodegenerative disorders as compared to amyloid-negative cognitively unimpaired controls, Parkinson’s disease and Alzheimer’s disease. Effect sizes (Hedges’s *g*) of different neurodegenerative disorders as compared to amyloid-negative cognitively unimpaired controls (**A** *n*  =  403), Parkinson’s disease (**B** *n*  =  311), and Alzheimer’s disease (**C** *n*  =  236). The bars represent the mean effect size for the cohort, whereas the error bars represent the standard deviation of effect size when considering the KCL and Lund cohorts separately. Those without error bars (e.g., VaD) are only included in one cohort. AD    Alzheimer’s disease (*n* = 236), ALS   amyotrophic lateral sclerosis (*n* = 50), CU Aβ− cognitively unimpaired without Aβ pathology (*n* = 403), CBS/PSP   corticobasal syndrome and progressive supranuclear palsy (*n* = 43), depression (*n* = 37), DS   Down syndrome (*n* = 29), DSAD   Down syndrome Alzheimer’s disease (*n* = 12), EOAD   early-onset Alzheimer’s disease (*n* = 82), FTD   frontotemporal dementia (*n* = 204), MCI Aβ− mild cognitive impairment without Aβ pathology (*n* = 170), MCI Aβ + mild cognitive impairment with Aβ pathology (*n* = 196), MSA   multiple system atrophy (*n* = 29), PD   Parkinson’s disease (*n* = 311), PDD/DLB   Parkinson’s disease dementia and dementia with Lewy bodies (*n* = 105), VaD vascular dementia (*n* = 22).

When combining the two cohorts, the largest effect sizes against CU Aβ− group were observed for DSAD (Hedges *g* = 1.87), MSA (Hedges *g* = 1.25), ALS (Hedges *g* = 1.19), CBS/PSP (Hedges *g* = 0.96), and FTD (Hedges *g* = 0.84). Medium effect sizes (Hedges *g* < 0.5) were observed for VaD and AD dementia (Fig. 2A). However, only small effect sizes existed in MCI groups (Hedges *g* < 0.1). When measuring the effect size of plasma NfL against the PD group (Fig. 2B), large effects sizes were observed for atypical parkinsonian disorders (CBS/PSP, Hedges *g* = 2.0; MSA, Hedges *g* = 1.4) and also large effect sizes for some cognitive impairment disorders (VaD, Hedges *g* = 1.88; FTD, Hedges *g* = 1.4; PDD/DLB, Hedges *g* = 1.1, AD dementia, Hedges *g* = 1.0). In contrast, only medium or small effect sizes were demonstrated when comparing AD dementia to other cognitive impairment disorders (Fig. 2C).

### Accuracy of plasma NfL in differentiating neurodegenerative disorders

**Fig. 3:**
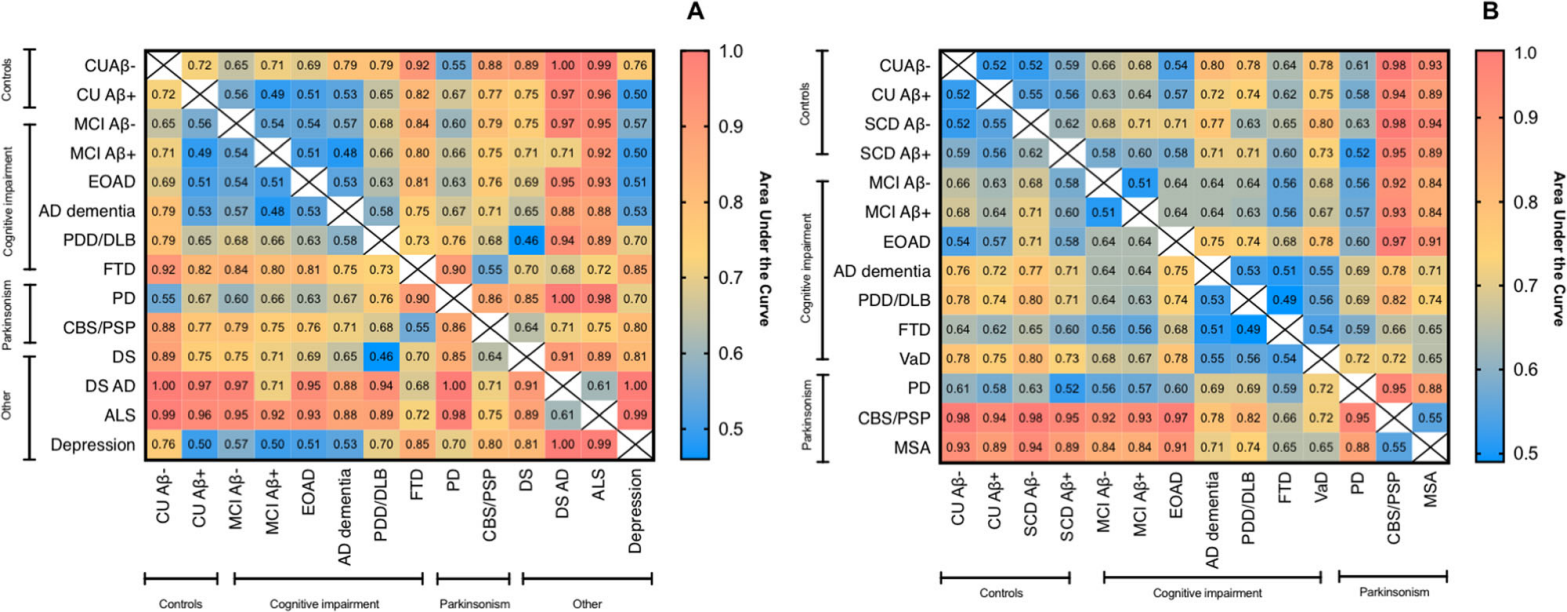
The diagnsotic accuracy of plasma NfL in neurodegenerative disorders. Heatmaps to demonstrate the accuracy (AUC) of plasma NfL to distinguish CU and neurodegenerative disorders in the KCL (**A**) and Lund (**B**) cohorts. Heatmaps tables that demonstrate sensitivity, specificity, and 95% CI of AUC displayed in the Supplementary Tables 4–5 and Supplementary Fig. 4. AD   Alzheimer’s disease, ALS   amyotrophic lateral sclerosis, CU Aβ− cognitively unimpaired without Aβ pathology, CU Aβ+ cognitively unimpaired with Aβ pathology, CBS/PSP   corticobasal syndrome and progressive supranuclear palsy, DS   Down syndrome, DSAD   Down syndrome Alzheimer’s disease, EOAD   early-onset Alzheimer’s disease, FTD   frontotemporal dementia, MCI Aβ− mild cognitive impairment without Aβ pathology, MCI Aβ+ mild cognitive impairment with Aβ pathology, MSA   multiple system atrophy, PD   Parkinson’s disease, PDD/DLB   Parkinson’s disease dementia and dementia with Lewy bodies, SCD Aβ− subjective cognitive decline without Aβ pathology, SCD Aβ+ subjective cognitive decline with Aβ pathology, VaD vascular dementia.

Next, we investigated the diagnostic accuracies of plasma NfL in differentiating among neurodegenerative disorders and also from CU groups. AUC values for the KCL and Lund cohorts are displayed in Fig. 3A and B, respectively. The 95% confidence intervals (CI) of AUC, sensitivity, and specificity estimates can be found in Supplementary Tables 5–6 and Supplementary Fig. 4.

As expected, ROC analyses for plasma NfL demonstrated low accuracy in separating CU Aβ− from CU Aβ+ , SCD, and MCI groups (AUC = 52–65%), but performed better for identifying AD dementia (KCL, AUC = 79%; Lund, AUC = 80%) with superior specificity (76–78%) than sensitivity (65–67%). High AUCs (>80%) were also found in distinguishing CU Aβ− from atypical parkinsonian in both cohorts and DS, DSAD, FTD, and ALS in the KCL cohort. Plasma NfL also performed well in identifying atypical parkinsonian disorders from PD patients with very high specificity in the KCL cohort (AUC = 86%; sensitivity = 56%; specificity = 89%) which was observed in the Lund cohort for both CBS/PSP (AUC = 95%; sensitivity = 51%; specificity = 100%) and MSA (AUC = 88%; sensitivity = 57%; specificity = 90%). Plasma NfL had a high accuracy in differentiating DS from DSAD (AUC = 91%; sensitivity = 100%; specificity = 71%). A moderate AUC in differentiating FTD from ALS (AUC = 72%) but higher for distinguishing FTD from depression (AUC = 85%) was observed. Low AUC’s were observed for differentiating AD dementia from other cognitive impairment disorders (e.g., VaD, PDD/DLB, FTD) and also PDD/DLB from atypical parkinsonian disorders.

**Fig. 4:**
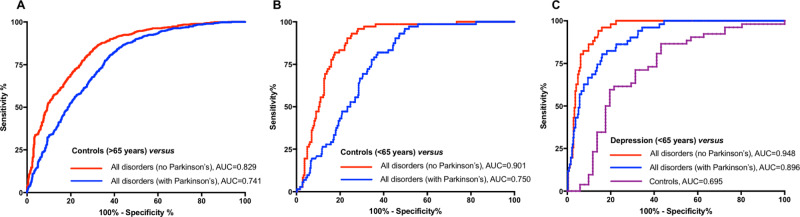
The diagnsotic accuracy of plasma NfL in identifying neurodegenerative disorders from controls (young/old) and depression. The performance of plasma neurofilament light (NfL) to identify neurodegenerative disorders from controls (CU and SCD) > 65 years of age (**A**), controls (CU and SCD) < 65 years of age (**B**), and depression (**C**).

In an additional analysis, combining the KCL and Lund cohorts, we investigated the diagnostic accuracy of plasma NfL to separate cognitively normal individuals (CU and SCD) from all neurodegenerative disorders (including MCI). This was performed in individuals >65 years (Fig. 4A) and <65 years (Fig. 4B) separately. We found that in individuals >65 years, plasma NfL had relatively good accuracy in identifying neurodegenerative disorders (irrespective of indiviudal diagnosis) from controls (AUC = 0.829, 95% CI, 0.82–0.86; Fig. 4A) but was less accurate if PD patients were included in the neurodegenerative disorder group (AUC = 0.74, 95% CI, 0.69–0.78; Fig. 4A). However, in individuals <65 years, plasma NfL demonstrated a higher accuracy in identifying neurodegenerative disorders from controls (AUC = 0.90, 95% CI, 0.86–0.93; Fig. 4B). Once more, adding PD patients into the neurodegenerative disorder group significantly reduced the diagnostic accuracy (AUC = 0.75, 95% CI, 0.70–0.78; Fig. 4B). Lastly, we compared the depression group in comparison to <65 years controls and neurodegenerative disorders (Fig. 4C). Plasma NfL exhibited high diagnostic performance in determining neurodegenerative disorders and depression (AUC = 0.948, 95% CI, 0.92–0.97), this remained highly accurate even when adding in PD patients (AUC = 0.896, 95% CI, 0.86–0.94).

### Concentration cutoff points for neurodegeneration using plasma NfL

Three cutoff points of plasma NfL concentration for neurodegeneration were applied (a) 90%, (b) 95%, and (c) 99% CI of CU Aβ− participants. Additional methods for cutoffs for plasma NfL were also derived by two other approaches (i) mean plus 2 standard deviations of the CU Aβ− participants and (ii) Gaussian mixture modeling. The cutoffs were performed and generated in the KCL cohort and then tested in the Lund cohort. The cutoff concentrations for all methods are reported in Supplementary Table 7.

**Fig. 5:**
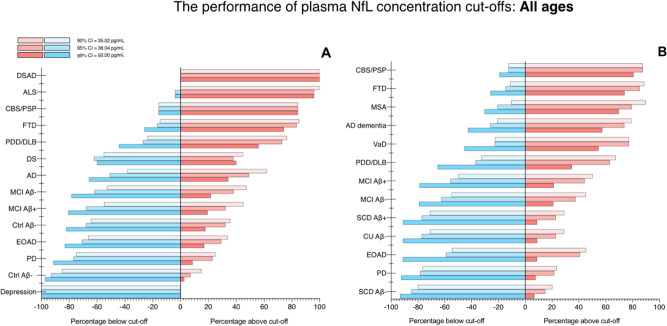
The performance of plasma NfL concentration cutoffs to identify neurodegenerative disorders of all ages. The performance of plasma neurofilament light (NfL) concentration cutoffs to identify neurodegenerative disorders in KCL (**A**) and Lund (**B**). AD   Alzheimer’s disease, ALS   amyotrophic lateral sclerosis, CBS   corticobasal syndrome, DLB   dementia with Lewy bodies, DS   Down syndrome, DSAD   Down syndrome Alzheimer’s disease, EOAD   early-onset Alzheimer’s disease, FTD   frontotemporal dementia. MCI   mild cognitive impairment, MSA   multiple system atrophy, PD   Parkinson’s disease, PDD   Parkinson’s disease dementia, PSP   progressive supranuclear palsy, SCD   subjective cognitive decline, VaD vascular dementia.

The performance of concentration cutoffs based on CI of CU Aβ− participants of all ages is demonstrated in Fig. 5. This method, which was derived in the KCL cohort, calculated plasma NfL concentration cutoffs at 35.02 pg/mL, 38.04 pg/mL, and 50.00 pg/mL for the 90%, 95%, and 99% CI of the CU Aβ− participants, respectively. In both the KCL and Lund cohorts, a more stringent cutoff (99% CI) demonstrated relatively low false positives for all CU groups and also for depression, PD and EOAD (0–12%). A more moderate cutoff (CI 90–95%) demonstrated higher percentages of false positives in the same groups (0–25%). On the other hand, the 99% CI cutoff failed to identify neurodegeneration with a high degree of accuracy in disease groups, whereas a 90% CI accurately classified >75% of participants with neurodegenerative disorders in the Lund cohort; VaD (77%), AD (79%), CBS/PSP (87%), FTD (88%), and MSA (89%). Similar findings were also observed in the KCL cohort, although the % abnormal for plasma NfL was lower for AD (68%) but higher for PDD/DLB (KCL = 78%; Lund = 68%). Concentration cutoffs of plasma NfL identified neurodegeneration in FTD (>75%), CBS/PSP (>80%), ALS (98–100%) and DSAD (100%) with very high accuracy. Plasma NfL cutoffs were then tested in ADNI participants (*n* = 870) to replicate the findings for AD dementia (Supplementary Fig. 5). Similar to the KCL and Lund cohorts, a 99% CI cutoff exhibited relatively low false positives in CU groups (<10%), whereas for AD dementia, a 90% CI cutoff correctly classified >75% of cases. Unlike the KCL and Lund cohorts, ADNI participants classified as MCI Aβ+ had a significantly higher (*P* < 0.001) percentage of individuals with abnormal NfL above a 90% CI cutoff (61%) than MCI Aβ− (49%).

Due to the strong relationship between age and NfL, age-related cutoffs were also determined (Supplementary Table 7). First, we tested >65 year cutoff, combining the KCL and Lund cohorts (*n* = 1646, Fig. 6A). As expected, the cutoff derived from CU participants aged 65+ yielded marginally higher plasma NfL cutoffs than previously described for 90%, 95%, and 99% CI-based approaches (37.02, 46.00, 54.80 pg/mL). While no major differences were observed from Fig. 5, lower percentages of abnormal plasma NfL were observed for Aβ− CU and SCD were lower (6%) as well as the PD group (7%) for the 99% CI cutoff as compared to the cutoff derived from all ages.

**Fig. 6:**
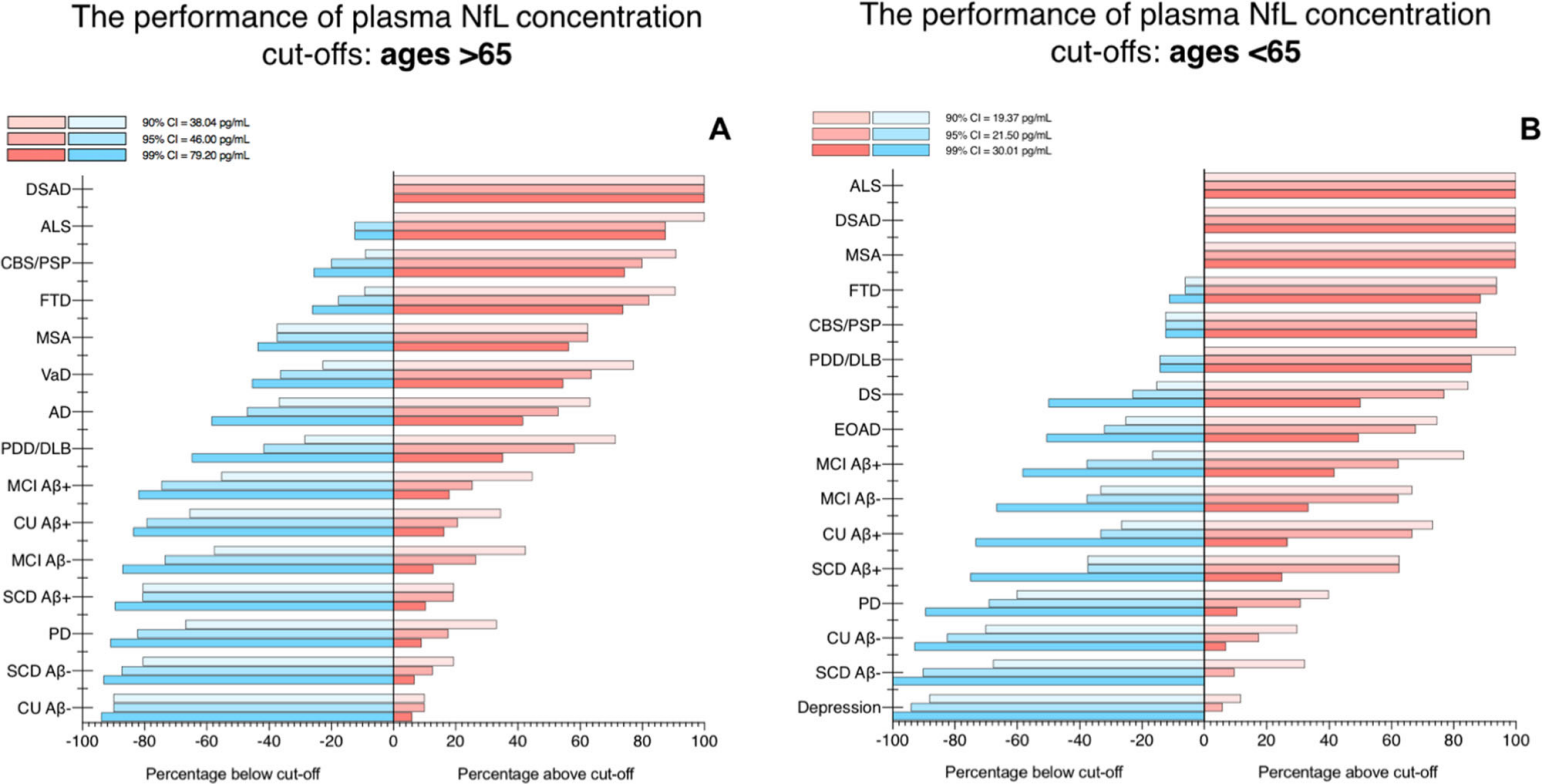
The performance of plasma NfL concentration cutoffs to identify neurodegenerative disorders in >65 and <65 years. The performance of plasma neurofilament light (NfL) concentration cutoffs to identify neurodegenerative disorders in >65 (**A**) and <65 (**B**). The KCL and Lund cohorts are combined for this analysis. AD   Alzheimer’s disease, ALS   amyotrophic lateral sclerosis, CBS   corticobasal syndrome, DLB   dementia with Lewy bodies, DS   Down syndrome, DSAD   Down syndrome Alzheimer’s disease, EOAD   early-onset Alzheimer’s disease, FTD   frontotemporal dementia. MCI    mild cognitive impairment, MSA   multiple system atrophy, PD   Parkinson’s disease, PDD   Parkinson’s disease dementia, PSP   progressive supranuclear palsy, SCD   subjective cognitive decline, VaD vascular dementia.

Concentration cutoffs in CU participants aged <65 were substantially lower; 19.37, 21.50, and 30.01 pg/mL, respectively, and were tested in participants in the KCL and Lund cohorts combined (*n* = 653, Fig. 6B). Firstly, with this age-related cutoff, abnormal levels of NfL were found in 100% of patients diagnosed with MSA, ALS, and DSAD regardless of % CI employed. Secondly, identifying abnormal NfL vastly improved in FTD (>90%), CBS/PSP (>90%), PDD/DLB (84%), and MCI groups (40–80%). While these improvements were seen for disorder groups, false positives for abnormal plasma NfL remained low for Aβ− controls (CU and SCD), depression, and PD (0–7%). Interestingly, higher rates of abnormal plasma NfL were now detected in CU and SCD that were Aβ+ (>22% in 90% CI;  >60% in 99% CI). Similarly, greater rates of abnormal plasma NfL were also observed in MCI Aβ+ compared with MCI Aβ−. Finally, improved rates of abnormal plasma NfL were observed in EOAD when utilizing an age-related cutoff (77%, 90% CI) which was comparable to abnormal NfL in AD using the >65-year cutoff. Interestingly, a small percentage (12%) of depression participants demonstrated abnormal plasma NfL when using an age-appropriate cutoff for this diagnostic group.

## Discussion

This study, to the best of our knowledge, includes the largest and most diverse investigation for plasma NfL comprising 2269 participants from CU individuals and 13 neurodegenerative disorders, Down syndrome, and depression. First, our findings corroborate, on a large scale, the globally increased plasma NfL concentration in major neurodegenerative disorders. Second, while these increases are seemingly not disease-specific, we demonstrate that plasma NfL is clinically useful in differentiating atypical parkinsonian disorders from PD, in identifying dementia in Down Syndrome, distinguishing neurodegenerative disorders from depression in older adults and, potentially, identifying frontotemporal dementia in patients with cognitive impairment. It was also apparent that the capabilities of plasma NfL to detect neurodegeneration were superior in younger (<65 years) than older (>65 years) individuals. However, NfL provides limited information in separating specific disorders of cognitive impairment (e.g., FTD vs AD), prodromal (e.g., CU vs SCD or MCI), or preclinical conditions (e.g., CU Aβ− vs CU Aβ+). Lastly, we derived data-driven and age-related concentration cutoffs that give relatively low false positives of abnormal plasma NfL but also indicate neurodegeneration in cortical neurodegenerative disorders, parkinsonian, and other neurogenerative disorders, depending on the cutoff strategy employed. The importance of age-related cutoffs was clearly demonstrated in disorders with a younger age of onset (e.g., EOAD, ALS, and FTD).

A recent meta-analysis on more than 10,000 individuals demonstrated that individuals with human immunodeficiency virus (HIV), FTD, ALS, and Huntington’s disease (HD) presented with CSF NfL concentrations averaging 21-fold, 11-fold, eightfold, and sixfold higher than CU controls, respectively^31^. In comparison, in the same study, CSF NfL was 1.9-fold higher in AD dementia patients. In the present plasma study, we also demonstrate that individuals with ALS and FTD presented with the highest concentrations of plasma NfL and among the largest effect sizes against CU individuals, albeit less dramatic than what has been reported for CSF. Although HIV and HD groups were not examined in this study, we were able to determine that DSAD and atypical parkinsonian disorders have the largest increases and effect sizes of plasma NfL as compared to individuals without cognitive impairment. The AD dementia population in this study was on average 1.8-fold higher than CU, mirroring the mild observations reported in CSF studies.

We tested the accuracy, sensitivity, and specificity of plasma NfL in differentiating neurodegenerative disorders. Although the majority of comparisons would not be a realistic diagnostic challenge in a clinical setting, high performance of plasma NfL was seen in predicting atypical parkinsonian disorders from PD. While plasma NfL data from atypical parkinsonian patients in the Lund cohort have been previously reported^26^, it is congruent with novel data included from the KCL cohort. In both cohorts, atypical parkinsonian disorders (e.g., CBS, PSP, MSA) had substantial increases in plasma NfL as compared to PD with very high diagnostic accuracies (KCL, AUC > 86%; Lund, AUC > 95%) and large effect sizes. Therefore, a presentation of parkinsonism with high levels of plasma NfL is highly suggestive of an atypical parkinsonian disorder and this finding is likely due to the degree of axonal damage being more severe in atypical parkinsonian disorders than in PD. Although not typically a diagnostic challenge, plasma NfL levels were able to distinguish ALS from controls in >90% of cases. In this study, we show the highest NfL levels of the thirteen neurodegenerative diseases that have been compared were observed in ALS and FTD. This may be indicative of the intensity of neurodegeneration or level of axonal damage and/or the extent of the degenerated axons. Substantial evidence supports that neuronal and axon damage in ALS and FTD results in the release of neurofilament proteins into the CSF and plasma^32,33^. Separately high levels of plasma NfL in ALS and FTD have also been linked to disease severity, as shown by NfL levels correlating with survival and disease progression in ALS and FTD^32,34,35^. Interestingly, ALS and FTD might be phenotypic extremes on a spectrum disorder, which is called motor neuron disease–FTD continuum, and up to 15% of all incidents in ALS cases are associated with FTD^36^. Yet, the diagnosis of FTD and especially the behavioral variant (bvFTD) subtype is often challenging, as the heterogeneous clinical manifestation may overlap not only with other neurodegenerative diseases but also with psychiatric disorders. A further novel contribution of this study is we demonstrate the normal plasma NfL concentrations of individuals with moderate and severe depression, and that high AUC (85%) existed when comparing depressed patients with those with an FTD diagnosis. In fact, plasma NfL had high accuracies in distinguishing moderate and severe depression from all neurodegenerative disorders (AUC = 0.95), and even when neurodegenerative disorders with typically lower concentrations were included (e.g., PD) (AUC = 0.89). Therefore, this study shows promise in plasma NfL discriminating between FTD (and other neurodegenerative disorders) and psychiatric disorders when the significant clinical overlap does exist^28^. Our data is also consistent with previous studies on plasma NfL in DS^37–39^ where an increase of plasma NfL levels was substantially higher in those with individuals with dementia. Using our defined concentration cutoffs, we were able to differentiate DSAD from DS in the KCL cohort (AUC = 0.91) and demonstrate that all DSAD patients exhibited abnormal plasma NfL when applying cutoffs.

In a novel approach, we derived and tested concentration cutoffs to identify neurodegeneration ranging from high specificity (99% CI) to a cutoff favoring greater sensitivity (90% CI)—this could be employed as a guide in primary care or memory clinic assessment. A plasma NfL cutoff using the 99% CI demonstrated the ability to give reliable low false positives in cognitively normal groups (e.g., CU, SCD) but also depression and PD groups were absent axonal damage is expected. We confirmed that NfL is abnormally elevated in multiple disorders but overlapping concentrations among disorders limit plasma NfL as a disease-specific marker. When a more sensitive cutoff was applied, abnormal NfL levels were consistently observed in the majority of neurodegenerative disorders. This also included AD dementia where plasma NfL is seen to be only mildly elevated as compared to other neurodegenerative disorders. These concentration cutoffs were independently tested in the ADNI cohort, in relation to the AD continuum, and produced similar results. However, recent data has clearly shown that plasma P-tau (either P-tau181^10,11,13^, P-tau217^18,40^, or P-tau231^19^) is better placed for AD diagnostics. Plasma P-tau can identify AD from non-AD dementias with high accuracy^10,18,19,35,41^ and correlates with the underlying pathogenesis^11,42^. Therefore, a positive plasma P-tau test would indicate AD. However, a negative P-tau test in combination with a positive NfL test would be highly supportive of non-AD dementia—ruling out AD, PD, and based on our data, primary psychiatric disorders as a diagnosis.

In addition to the diagnostic capabilities of plasma NfL, this study highlights other key factors which should be detailed. Multiple lines of evidence have reported age and CSF NfL as having strong relationships with plasma NfL. While these statements are without-a-doubt true, based on the findings presented herein one cannot simply apply this generalized rule to all age groups and conditions. First, plasma NfL is unequivocally influenced by age but this association is stronger in younger individuals (e.g., <65 years) and, to some degree, the relationship is diminished in older individuals (e.g., >65 years, Supplementary Table 2). This is due to older individuals being more likely to have developed a neurodegenerative condition and these disorders have a different relationship with age; that is, neurodegenerative disorders that typically exhibit higher concentrations of plasma NfL have weaker correlations with age (e.g., FTD). Furthermore, plasma NfL is likely to increase in response to pathologies that manifest in later life (e.g., limbic-predominant age-related TDP-43 encephalopathy, LATE). In our study, the influence of age on NfL is shown in multiple aspects, but most prominently by EOAD patients seemingly being no different from CU adults if no age adjustment is taken into consideration. Our <65-year plasma NfL cutoffs (19.4, 21.5, 30.0 pg/mL) were substantially lower as to compared older cutoffs (38.0, 46.0, 79.20 pg/mL) and when this was applied, EOAD patients had the equivalent rate of abnormal plasma NfL as typical late-onset AD dementia (Fig. 6)—consistent with the reported literature on familial AD^43,44^. Neurodegenerative disorders with a typically younger age of onset also demonstrated higher rates of abnormal NfL if a < 65-year cutoff was applied (e.g., FTD, MSA) and plasma NfL was shown to be better at identifying neurodegenerative disorders in younger individuals (<65 years) in comparisons to older individuals (Fig. 4). We also observed that age-related cutoffs may be more sensitive to neurodegeneration related to Aβ deposition, although it is clear that recent developments in plasma p-tau181, p-tau217 and p-tau231 would be a superior measures of Aβ and tau pathologies^10,11,13,14,19,35,41^, as previously mentioned. In individuals <65 years, rates of abnormal plasma NfL were threefold higher in Aβ+ controls as compared to Aβ- controls and also higher in MCI Aβ+ than MCI Aβ−. The influence of Aβ positivity on plasma NfL has been previously described^22,45–47^ however, in our study, this was far more apparent in the younger age groups. It is not guaranteed that Aβ deposition leads to cognitive decline nor is there a linear relationship between Aβ burden and the extent of cognitive impairment; however, when coupled to age-dependent abnormal levels of NfL (a proxy for on-going axonal damage), this may indicate those at a far greater risk. This is further supported by the very low rate of false positives of plasma NfL in Aβ− controls but also in patients with depression and PD which are likely to be Aβ−. We have also demonstrated that the plasma-to-CSF relationship of NfL is dependent on the condition. While the majority of cognitive impairment disorders and parkinsonian disorders display a strong relationship between plasma and CSF NfL, VaD, and CBS/PSP have a nonsignificant and weak relationship (Supplementary Results 3). This is an important consideration when using plasma NfL to infer CSF NfL levels.

Our study has limitations. Although this study was performed in 2269 individuals, in certain diagnostic categories and comparisons, it was underpowered. Several neurodegenerative diseases included in this study, such as DS and atypical parkinsonian disorders have a relatively small number of participants. However, although our sample size was small in these groups, we were able to show with excellent accuracy and effect sizes the differentiation between CU and disorders but also within neurodegenerative disorders which may be clinically challenging. In this study, no neuropathological confirmation of any individual was available and only Aβ burden was indexed in CU, SCD, MCI, and AD. Therefore, despite being very well clinically characterized, there may be some diagnostic uncertainty attributed the non-AD dementias. Furthermore, we do acknowledge that a number of some of the disorders included in this study are likely to be at an advanced disease stage given that a clinical diagnosis had been assigned—therefore, we cannot determine how well NfL identifies these disorders in the earlier stages of the disease. Unlike many putative plasma biomarkers that have preceded it measurements of plasma NfL are robust. In this study, we have technically demonstrated a very high correlation in the measurements of plasma NfL using two different assays on the Simoa platform, which were performed in independent laboratories. However, it must be noted that absolute concentrations of plasma NfL differed between assays and therefore platform dependent cutoffs would need to be calculated in the likelihood of multiple methodologies to measure NfL in blood in the future. Despite being a multicenter study, this has not influenced our results. This has been shown by (i) the very high level of replication between the two cohorts, even when applying a concentration derived in KCL and tested in Lund and (ii) CU participants provided by multiple centers having similar concentrations of plasma NfL despite varying preanalytical procedures.

In conclusion, in two large independent datasets, we have detailed the meaningful strengths and weaknesses of utilizing plasma NfL as a biomarker for neurodegeneration that could be useful in a primary care setting. Plasma NfL concentrations are increased across multiple neurodegenerative disorders but are highest in samples from individuals with ALS, FTD, and DSAD. Though plasma NfL cannot differentiate between different cognitive impairment disorders, in patients with parkinsonism, high plasma NfL values indicate atypical parkinsonian disorders and in patients with DS, high plasma NfL differentiates between those with and without dementia, suggesting it may be useful in both clinical and research settings in these patients. Furthermore, plasma NfL can differentiate between moderate/severe depression from neurogenerative disorders, which has direct implications for many disorders e.g., FTD. Data-driven age-related concentration cutoffs suggested in this work demonstrated that plasma NfL is suitable to identify neurodegeneration in many neurodegenerative disorders, though false positives rates were low when using an age-appropriate cutoff set using the 99% CI of Aβ− CU.

## Methods

### Study participants

In this study, 2269 individuals from two multicentre cohorts were included. The KCL cohort represents a multicenter collection of participants (*n* = 805) collated at the Maurice Wohl Clinical Neuroscience Institute, King’s College London^5,48–60^. This consisted of CU individuals (*n* = 158), mild cognitive impairment (MCI, *n* = 86), early-onset Alzheimer’s disease (EOAD < 65 years, *n* = 59), AD dementia (*n* = 102), FTD (*n* = 54), PD (*n* = 140), Parkinson’s disease dementia and dementia with Lewy bodies (PDD/DLB, *n* = 59), corticobasal syndrome and progressive supranuclear palsy (CBS/PSP, *n* = 19), Down Syndrome (DS, *n* = 41; 12 with dementia), amyotrophic lateral sclerosis (ALS, *n* = 50), and depression (HAM-D > 13, *n* = 37).

The Lund cohort consisted of 1464 participants enrolled as part of the prospective and longitudinal Swedish BioFINDER study (clinical trial no. NCT01208675) which recruited at the Neurology and Memory Clinics, Skåne University Hospital, Lund, Sweden, between 2008 and 2014^61,62^. In addition, FTD cases were obtained from the Erasmus Medical Centre, Rotterdam, The Netherlands^63^ and Lund Prospective Frontotemporal Dementia Study (LUPROFS)^64^. The Lund cohort included CU (*n* = 376), subjective cognitive decline (SCD, *n* = 209), and seven diagnostic groups in common with the KCL cohort (MCI, *n* = 280; EOAD < 65 years, *n* = 23; AD dementia, *n* = 134; FTD, *n* = 150; PD, *n* = 171; PDD/DLB, *n* = 46; CBS/PSP, *n* = 24). In addition, the Lund cohort included patients with multiple system atrophy (MSA, *n* = 29) and vascular dementia (VaD, *n* = 22). The inclusion criteria for CU individuals in the Lund cohort have been previously detailed^11^. In brief, CU individuals must fulfill these criteria: (1) absence of cognitive symptoms as assessed by a physician with a special interest in cognitive disorders; (2) Mini-Mental State Examination (MMSE) 28–30 at screening visit; (3) did not fulfill the criteria for MCI or any dementia disorder. The exclusion criteria included (1) significant unstable systemic illness that made it difficult to participate in the study; (2) current significant alcohol or substance misuse; and (3) significant neurological or psychiatric illness. The inclusion criteria for patients with SCD or MCI (defined using criteria by Petersen^65^) were (1) referred to a participating memory clinic because of cognitive complaints; (2) did not fulfill the criteria for any dementia disorder. The exclusion criteria were the same as the for the CU participants with the addition of (1) cognitive impairment that, without doubt, could be explained by other specific non-neurodegenerative disorders, such as brain tumor or subdural hematoma. The KCL cohort, where possible, followed the same inclusion and exclusion criteria for CU and MCI participants^49,52,53,58,60,66,67^ and is further detailed in Supplementary Table 1. No SCD participants were included in the KCL cohort.

The Lund (BioFINDER study) cohort was approved by the Regional Ethics Committee in Lund, Sweden (case number 2014/223) and all participants gave written informed consent to participate in the study. The KCL cohort was approved by the appropriate Regional Ethics Committees (Supplementary Table 1) and all participant gave their informed consent.

To confirm findings related to the AD continuum, this study also obtained data from the Alzheimer’s disease Neuroimaging Initiative (ADNI) database (clinical trial no. NCT00106899; adni.loni.usc.edu) for 870 individuals (CU, *n* = 290; MCI, *n* = 442; AD dementia, *n* = 138). AD dementia participants had a Mini-Mental State Examination (MMSE) ranging between 20 and 26; Clinical Dementia Rating (CDR) 1 or above and met criteria for probable AD according to the National Institute of Neurological and Communicative Disorders and Stroke–Alzheimer’s Disease and Related Disorders Association (NINCDS-ADRDA). Participants were classified as MCI if MMSE ranged between 24 and 30, CDR 0.5 (with the memory box score being 0.5 or greater), and did not meet the criteria for dementia according to the NINCDS-ADRDA.

### Determination of amyloid-β status

Individuals clinically classified as CU, SCD (Lund cohort only), and MCI were further categorized into Aβ-negative (Aβ−) or Aβ-positive (Aβ+ ). In the KCL cohort, Aβ cutoff values for assigning positivity were determined by CSF Aβ42, [^11^C]PiB‐PET, or [^18^F]AZD4694 as outlined in Supplementary Table 1. It was determined that 28/158 and 31/86 of CU and MCI were Aβ+ , respectively. In the Lund cohort, Aβ-positivity was classified by CSF with Aβ42/Aβ40 < 0.091 by EUROIMMUN immunoassays (EUROIMMUN AG, Lübeck, Germany)^68^. This determined that 103/376, 75/209, and 165/280 of CU, SCD, and MCI individuals were Aβ + , respectively. For ADNI, brain Aβ load—at the last available visit of each subject—was estimated using [^18^F]florbetapir PET. The cutoff to determine Aβ-positivity was 1.11 SUVR, as suggested in the ADNI protocol. According to this criterion, 100/290 and 247/442 CU and MCI were Aβ-positive, respectively.

### Biochemical analysis

Blood sampling procedures for cohorts included in the KCL and Lund cohorts are summarized in Supplementary Table 1. Blood collection and processing procedures for ADNI have been detailed elsewhere^22^. Plasma NfL concentration was measured using two highly correlated versions of a single-molecule array (Simoa; Quanterix; Billerica, MA) method. For the KCL cohort, the commercially available NF-light assay was utilized (NF-light™ # 103186) and all samples were analyzed at the Maurice Wohl Clinical Neuroscience Institute, King’s College London, UK. Data acquisition spanned seventeen analytical runs and all the samples were above the lower limit of quantification reported for this assay (LLOQ, 0.174 pg/mL). For the low-concentration control sample (8.5 pg/mL), the intra-assay coefficient of variation was 7.5% and the inter-assay coefficient of variation was 12.8%, while for the high-concentration quality control sample (112 pg/mL), the corresponding coefficients of variation were 9.5% and 13.8%, respectively. For the Lund and ADNI cohorts, an in-house Simoa assay, utilizing the same antibodies and calibrator as the commercial kit^20^, and was performed at the Clinical Neurochemistry Laboratory, University of Gothenburg, Sweden. For the Lund cohort, data acquisition spanned twenty-three analytical runs and all the samples were above the lower limit of quantification (6.7 pg/mL). For the low-concentration control sample (12.2 pg/mL), the intra-assay coefficient of variation was 5.5% and the inter-assay coefficient of variation was 8.2%, whilee for the high-concentration quality control sample (107.3 pg/mL), the corresponding coefficients of variation were 9.3% and 9.4%, respectively. Data acquisition methods for NfL measurements in ADNI have been previously described^21,22^.

### Harmonization of KCL and Lund cohorts

Quality control (QC) samples provided by the Lund cohort (*n* = 30) were quantified at random in the KCL analysis. High concordance (*r* = 0.925, *P* < 0.0001, Supplementary Fig. 1A) was achieved between the QC samples despite the absolute values in the KCL cohort being significantly higher (*P* = 0.025, Supplementary Fig. 1B). Based on this QC data, a correction factor of 1.18 was applied to all Lund and ADNI samples to adjust the data to the KCL cohort for all subsequent analyses.

### Statistical analysis

Associations between continuous variables were tested with Spearman’s rank-order correlation with a partial correlation adjusting for age. Group differences were assessed by Mann–Whitney test or one-way Kruskal–Wallis test by ranks, with post hoc Dunn’s test where appropriate. To measure the specificity and sensitivity of plasma NfL, we calculated the AUC of the receiver operating characteristics (ROC) using the “AUC” and “pROC” packages for R. Cutoff concentrations for plasma NfL were defined in the KCL cohort and three variations were investigated a) 90%, 95% and 99% confidence interval of CU Aβ−, b) mean plus 2 standard deviations of the CU Aβ− and c) Gaussian mixture modeling (GMM). Hedges’ *g* statistical unit was used to report the effect size. SPSS (IBM, Armonk, NY) and the R programming language (version 3.4.3) were used for statistical analysis and Graph Pad PRISM for data visualization.

### Reporting summary

Further information on research design is available in the Nature Research Reporting Summary linked to this article.

## Supplementary information

Supplementary Information

Reporting summary

## Data Availability

The Source file gives raw plasma NfL values for the KCL, Lund, and ADNI cohorts. Anonymized data will be shared by request from a qualified academic investigator for the sole purpose of replicating procedures and results presented in the article and as long as data transfer agrees with EU legislation on the general data protection regulation and decisions by the Ethical Review Board of the appropriate cohorts, which should be regulated in a material transfer agreement. The ADNI data was accessed and is available via adni.loni.usc.edu. Source data are provided with this paper.

## Source data

Source Data
