# Supplementary material for: A multicentre validation study of the diagnostic value of plasma neurofilament light
Source: Nat Commun. 2021 Jun 7;12:3400. doi: 10.1038/s41467-021-23620-z (PMC8185001; doi:10.1038/s41467-021-23620-z)
Supplement: Supplementary file 1 — Supplementary Information [file 41467_2021_23620_MOESM1_ESM.pdf]

Supplementary Information

A multicentre validation study of the diagnostic value of plasma neurofilament light:

Ashton NJ et al.

Supplementary Table 1. Diagnostic and plasma EDTA sampling criteria for the KCL & Lund cohorts

Supplementary Table 2. Spearman Rank Correlations between age and plasma NfL separated by diagnostic groups

Supplementary Table 3. The association of plasma NfL with sex depending on diagnostic category

Supplementary Table 4. Spearman Rank Correlations between disease severity and plasma NfL separated by diagnostic groups.

Supplementary Table 5. Accuracy (AUC) 95% confidence interval (CI) of plasma NfL to differentiate healthy controls, neurodegenerative and neurological disorders in the KCL cohort

Supplementary Table 6. Accuracy (AUC) 95% confidence interval (CI) of plasma NfL to differentiate healthy controls, neurodegenerative and neurological disorders in the Lund cohort

Supplementary Table 7. Concentration cut-offs for plasma neurofilament light calculate in the KCL cohort

Supplementary Results 1. Demographics of the KCL and Lund cohorts

Supplementary Results 2. Correlations of plasma and cerebrospinal fluid (CSF) NfL

Supplementary Figure 1. Comparison of quality control (QC) samples between KCL and Lund cohorts**.**

Supplementary Figure 2. The correlations of plasma NfL and age in the KCL (A) and Lund (B) cohorts.

Supplementary Figure 3. Correlations of plasma NfL and CSF NfL in the Lund cohort unadjusted for age.

Supplementary Figure 4. The sensitivity and specificities of plasma NfL to differentiate healthy controls, neurodegenerative and neurological disorders in the KCL (A) and Lund (B) cohorts

Supplementary Figure 5. The performance of plasma neurofilament light (NfL) concentration cut-offs to identify neurodegenerative disorders in ADNI.

Supplementary References

Supplementary Table 1. Diagnostic and plasma EDTA sampling criteria of the KCL & Lund cohorts

Study Cohort	Centre	Cohort design	Diagnostic groups	Clinical Criteria	Aβ status	EDTA plasma procedure
King's College London (KCL) Cohort	Baltimore Longitudinal Study of ageing (BLSA)[1]	Prospective, longitudinal, multicenter	Cognitively unimpaired (Aβ-/Aβ+)	N/A	[^{11}C]PiB PET	Fasting, <4-hours, +4˚C, -80°C storage
	Brescia DMA Study Biobanking[2]	Prospective, longitudinal	Cognitively unimpaired (Aβ-/Aβ+); Parkinson's disease; Parkinson disease dementia; Dementia with Lewy bodies	Postuma RB[3] Emre M[4] McKeith IG[5]	CSF Aβ42 <550 pg/mL	Fasting, <2-hours, 3000 *g*, 20 min +4˚C, -80°C storage
	NEURODEM study, Chieti	Prospective, longitudinal	Parkinson's disease; Parkinson disease dementia; Dementia with Lewy bodies	Hughes AJ[6] McKeith IG[5]	N/A	Fasting, <2-hours, 2000 *g*, 10 min +4˚C, -80°C storage
	Dementia Case Register (DCR)[7]	Prospective and longitudinal,	Alzheimer's disease	McKhann G[8]	N/A	Fasting, <2-hours, 3000 *g*, 8 min +4˚C, -80°C storage
	Biodonostia Health Research Institute biobank[9]	Prospective and longitudinal,	Amyotrophic lateral sclerosis	El Escorial criteria[10] King's Staging system[11]	N/A	Fasting, <2-hours, 1500g, 15 min +4˚C, -80°C storage
	European Medical Information Framework (EMIF) 500 study[12] (Barcelona St Pau[13], Milan[14], Perguia[15])	Prospective, longitudinal, and multicenter	Alzheimer's disease; Cognitively unimpaired (Aβ-/Aβ+); Early onset Alzheimer's disease; Mild cognitive impairment (Aβ-/Aβ+)	McKhann G[8] Petersen RC[16]	Barcelona, CSF Aβ42 <550 pg/mL; Perguia, CSF Aβ42 <800 pg/mL; CSF Milan, Aβ42 <600 pg/mL	Fasting, <2-hours, 3000 rpm, 8 min +4˚C, -80°C storage
	Young Onset Dementia Assessment Service (YODAS), Leicestershire Partnership National Health Service (NHS) Trust[17,18]	Prospective, longitudinal, and multicenter	Early onset Alzheimer's disease	McKhann G[8]	N/A	Fasting, <2-hours, 3000 *g*, 8 min +4˚C, -80°C storage
	Karolinska/Stockholm[19]	Prospective, longitudinal, and multicenter	Parkinson's disease	Gelb DJ[20]	N/A	Fasting, <30-minutes, 800 *g*, 20 min +4˚C, -80°C storage

	LonDownS[21]	Observational, and longitudinal	Down syndrome; Down syndrome with Alzheimer's disease	Sheehan R[22]	N/A	Non-fasting, <3-hours, 2200 *g*, 10 min +4˚C, -80°C storage
	Translational Biomarkers in Aging and Dementia (TRIAD)[23]	Observational, and longitudinal	Cognitively unimpaired (Aβ-/Aβ+); Early onset Alzheimer's disease; Frontotemporal dementia	Snowden JS[24]<br>McKhann G[25]<br>Neary D[26]	[18F]AZD4694 (visual rating)	Fasting, <2-hours, 2200 *g*, 10 min RT, -80°C storage
	Kings College London, BIODEP (BIOmarkers in DEPression) study[27]	Observational, and longitudinal	Depression	DSM-5	N/A	Fasting, <2-hours, 1600 *g*, 15 min RT, -80°C storage
	University in San Francisco (UCSF)[28]	Prospective and longitudinal	Alzheimer's disease; Cortical basal syndrome; Progressive supranuclear palsy; Cognitively unimpaired (Aβ-/Aβ+); Early onset Alzheimer's disease; Frontotemporal dementia	McKhann G[25]<br>Neary D[26]<br>Petersen[16]	[11C]PiB PET	Fasting, <2-hours, 2000 *g*, 10 min +4˚C, -80°C storage
**Lund Cohort**	Swedish BioFINDER[29]	Prospective and longitudinal	Alzheimer's disease; Cortical basal syndrome; Progressive supranuclear palsy; Cognitively unimpaired (Aβ-/Aβ+); Early onset Alzheimer's disease; Mild cognitive impairment (Aβ-/Aβ+); Multiple system atrophy; Parkinson's disease; Parkinson disease dementia; Dementia with Lewy bodies; Subjective cognitive decline (Aβ-/ Aβ+)	McKhann G[25]<br>Rascovsky K[30]<br>Gelb DJ[20]<br>Gilman S [31]<br>Litvan I [32]<br>Litvan I [33]	CSF Aβ42/Aβ40 <0.091	Fasting, <1-hour, 2000 *g*, 10 min +4˚C, -80°C storage
	Lund Prospective Frontotemporal Dementia Study (LUPROFS)[34]	Prospective and longitudinal	Frontotemporal dementia	Rascovsky K[30]	N/A	Fasting, <1-hour, 2000 *g*, 10 min +4˚C, -80°C storage
	Erasmus Medical Centre, Rotterdam[35]	Prospective and longitudinal	Alzheimer's disease; Early onset Alzheimer's disease; Frontotemporal dementia	McKhann G[25]<br>Neary D[26]	N/A	Fasting, <1-hour, 2000 *g*, 10 min +4˚C, -80°C storage

Supplementary Table 2. Spearman Rank Correlations between age and plasma NfL separated by diagnostic groups.

Diagnostic group	Lund Cohort		KCL Cohort	
	r	*P* value	r	*P* value
**All participants**	**0.411**	**<0.0001**	**0.393**	**<0.0001**
**>65 years**			**0.191**	**<0.0001**
**<65 years**			**0.354**	**<0.0001**
CU Aβ-	0.491	<0.0001	0.450	<0.0001
CU Aβ+	0.479	<0.0001	0.540	<0.0001
SCD Aβ-	0.587	<0.0001	NA	NA
SCD Aβ+	0.327	<0.0001	NA	NA
MCI Aβ-	0.356	<0.0001	0.339	0.010
MCI Aβ+	0.217	0.005	0.072	ns
EOAD	0.694	ns	0.166	ns
AD dementia	0.392	<0.0001	0.356	<0.0001
FTD	0.247	0.005	0.175	0.025
PD	0.591	<0.0001	0.574	<0.0001
PDD/DLB	0.480	<0.0001	0.356	0.001
CBS/PSP	0.343	ns	0.120	ns
MSA	0.535	0.010	NA	NA
VaD	-0.113	ns	NA	NA
DS (all)	NA	NA	0.658	<0.0001
ALS	NA	NA	0.058	ns
Depression	NA	NA	0.331	0.018

Supplementary Table 3. The association of plasma NfL with sex depending on diagnostic category**.** P values (unadjusted for multiple comparisons) are from unpaired *t* test. F = female, M = male.

**Diagnostic group**	**Lund Cohort**				**KCL Cohort**			
	F, mean (SD)	M, mean (SD)	*t*	*P* value	F, mean (SD)	M, mean (SD)	*t*	*P* value
**All participants**	**33.8 (28.7)**	**31.0 (30.8)**	**1.6**	**ns**	**31.8 (34.3)**	**37.0 (42.7)**	**-2.0**	**0.05**
CU Aβ-	22.5 (13.0)	21.2 (10.3)	0.9	ns	17.4 (8.5)	16.2 (8.0)	0.8	ns
CU Aβ+	35.4 (89.3)	24.0 (11.5)	0.8	ns	29.0 (27.1)	31.1 (19.6)	-0.3	ns
SCD Aβ-	18.2 (10.0)	19.9 (10.7)	-0.9	ns	NA			
SCD Aβ+	23.0 (13.2)	25.1 (10.8)	-0.7	ns	NA			
MCI Aβ-	27.9 (14.9)	28.1 (31.9)	-0.0	ns	25.1 (13.0)	31.4 (19.7)	-1.4	ns
MCI Aβ+	28.8 (19.0)	30.2 (16.0	-0.5	ns	24.6 (7.1)	44.8 (45.5)	-1.8	ns
EOAD	25.5 (9.7)	22.0 (8.6)	0.87	ns	23.2 (16.0)	25.0 (18.2)	-0.4	ns
AD dementia	47.0 (31.7)	42.1 (23.3)	1.0	ns	40.9 (5.9)	37.0 (8.8)	0.6	ns
FTD	71.1 (49.0)	62.2 (63.4)	1.0	ns	61.4 (28.2)	59.0 (49.0	0.2	ns
PD	23.8 (21.2)	18.6 (13.4)	1.8	ns	20.6 (10.8)	20.4 (10.9)	0.2	ns
PDD/DLB	42.0 (33.7)	42.1 (46.1)	-0.0	ns	45.2 (24.2)	36.2 (15.4)	1.6	ns
CBS/PSP	45.8 (12.5)	53.7 (27.0)	-1.0	ns	59.6 (27.5)	48.6 (30.7)	0.8	ns
MSA	43.1 (26.7)	47.6 (28.8)	-0.4	ns	NA			
VaD	52.8 (21.2)	49.6 (23.7)	0.8	ns	NA			
DS	NA				29.2 (18.1)	30.9 (20.9)	-0.2	ns
DS AD	NA				66.8 (12.8)	67.9 (8.7)	-0.1	ns
ALS	NA				102 (117)	113 (88.6)	-0.4	ns
MS	NA				31.3 (36.5)	23.6 (22.8)	0.8	ns
Depression	NA				8.7 (4.6)	7.7 (3.8)	0.8	ns

Supplementary Table 4. The association of plasma NfL with disease severity. Data are Spearman correlation coefficients (P value) with significant results shown in bold.

Diagnostic group	Lund Cohort		KCL Cohort	
	r	*P* value	r	*P* value
**MCI all**				
MMSE	-0.072	ns	-0.055	ns
**MCI Aβ-**				
MMSE	0.088	ns	0.044	ns
**MCI Aβ+**				
MMSE	-0.145	ns	-0.101	ns
**EOAD**				
MMSE	-0.02	ns	-0.197	ns
**AD dementia**				
MMSE	0.015	ns	-0.022	ns
**FTD**				
MMSE	-0.078	ns	-0.095	ns
**PD**				
**UPDRS-III**	**0.240**	**0.003**	**0.344**	**<0.001**
**Hoehn & Yehr**	**0.258**	**0.002**	**0.353**	**<0.001**
**PDD/DLB**				
UPDRS-III	0.227	ns	0.127	ns
Hoehn & Yehr	0.359	ns	0.220	ns
MMSE	-0.026	ns	-0.203	ns
**CBS/PSP**				
UPDRS-III	0.318	ns	0.345	ns
Hoehn & Yehr	0.360	ns	0.398	ns
**MSA**				
**UPDRS-III**	**0.653**	**<0.001**	NA	NA
Hoehn & Yehr	0.344	ns	NA	NA
**VaD**				
MMSE	-0.114	ns	NA	NA
**ALS**				
Diagnostic delay	NA	NA	0.152	ns
Disease duration			0.039	ns
**Down syndrome**				
CAMDEX-DS*			**0.487**	**0.003**

* memory & orientation domains only

Supplementary Table 5. Accuracy (AUC) 95% confidence interval (CI) of plasma NfL to differentiate healthy controls, neurodegenerative and neurological disorders in the KCL cohort

CI 95%	Ctrl Aβ-	Ctrl Aβ+	MCI Aβ-	MCI Aβ+	EOAD	AD Dementia	FTD	PD	PDD/DLB	CBS/PSP	DS	DS AD	ALS	Depression
CUAβ-		0.60-0.83	0.56-0.74	0.59-0.81	0.60-0.76	0.63-0.77	0.88-0.96	0.48-0.62	0.71-0.87	0.77-0.98	0.81-0.96	0.99-1	0.98-0.99	0.68-0.83
CU Aβ+	0.60-0.83		0.42-0.68	0.33-0.63	0.38-0.63	0.41-0.64	0.72-0.91	0.55-0.78	0.52-0.77	0.61-0.92	0.61-0.88	0.92-1	0.91-0.99	0.35-0.65
MCI Aβ-	0.56-0.74	0.42-0.68		0.41-0.67	0.43-0.64	0.76-0.91	0.76-0.91	0.50-0.69	0.57-0.77	0.65-0.92	0.64-0.86	0.92-1	0.91-0.98	0.45-0.68
MCI Aβ+	0.59-0.81	0.33-0.63	0.41-0.67		0.38-0.63	0.36-0.58	0.69-0.89	0.54-0.76	0.54-0.76	0.59-0.89	0.57-0.84	0.57-0.84	0.84-0.99	0.35-0.65
EOAD	0.60-0.76	0.38-0.63	0.43-0.64	0.38-0.63		0.44-0.62	0.73-0.88	0.54-0.72	0.52-0.72	0.61-0.89	0.57-0.79	0.90-0.99	0.89-0.97	0.39-0.61
AD Dementia	0.63-0.77	0.41-0.64	0.76-0.91	0.36-0.58	0.44-0.62		0.67-0.82	0.59-0.73	0.49-0.67	0.58-0.84	0.55-0.74	0.81-0.94	0.83-0.93	0.44-0.62
FTD	0.88-0.96	0.72-0.91	0.76-0.91	0.69-0.89	0.73-0.88	0.67-0.82		0.85-0.95	0.63-0.82	0.39-0.70	0.58-0.81	0.54-0.81	0.62-0.81	0.76-0.92
PD	0.48-0.62	0.55-0.78	0.50-0.69	0.54-0.76	0.54-0.72	0.59-0.73	0.85-0.95		0.67-0.84	0.75-0.97	0.76-0.92	0.99-1	0.96-0.99	0.62-0.77
PDD/DLB	0.71-0.87	0.52-0.77	0.57-0.77	0.54-0.76	0.52-0.72	0.49-0.67	0.63-0.82	0.67-0.84		0.52-0.83	0.33-0.58	0.87-0.99	0.82-0.95	0.59-0.80
CBS/PSP	0.77-0.98	0.61-0.92	0.65-0.92	0.59-0.89	0.61-0.89	0.58-0.84	0.39-0.70	0.75-0.97	0.52-0.83		0.45-0.82	0.53-0.89	0.62-0.88	0.63-0.95
DS	0.81-0.96	0.61-0.88	0.64-0.86	0.57-0.84	0.57-0.79	0.55-0.74	0.58-0.81	0.76-0.92	0.33-0.58	0.45-0.82		0.82-0.99	0.81-0.95	0.69-0.91
DS AD	0.99-1	0.92-1	0.92-1	0.57-0.84	0.90-0.99	0.81-0.94	0.54-0.81	0.99-1	0.87-0.99	0.53-0.89	0.82-0.99		0.45-0.75	0.99-1
ALS	0.98-0.99	0.91-0.99	0.91-0.98	0.84-0.99	0.89-0.97	0.83-0.93	0.62-0.81	0.96-0.99	0.82-0.95	0.62-0.88	0.81-0.95	0.45-0.75		0.97-1
Depression	0.68-0.83	0.35-0.65	0.45-0.68	0.35-0.65	0.39-0.61	0.44-0.62	0.76-0.92	0.62-0.77	0.59-0.80	0.63-0.95	0.69-0.91	0.99-1	0.97-1	

Supplementary Table 6. Accuracy (AUC) 95% confidence interval (CI) of plasma NfL to differentiate healthy controls, neurodegenerative and neurological disorders in the Lund cohort

CI 95%	CUAβ-	CU Aβ+	SCD Aβ-	SCD Aβ+	MCI Aβ-	MCI Aβ+	EOAD	AD Dementia	FTD	PD	PDD/DLB	CBS/PSP	MSA	VaD
CUAβ-		0.45-0.59	0.46-0.58	0.51-0.66	0.59-0.71	0.62-0.72	0.36-0.71	0.70-0.81	0.56-0.70	0.55-0.66	0.41-0.68	0.94-1	0.87-0.97	0.65-0.91
CU Aβ+	0.45-0.59		0.47-0.62	0.47-0.64	0.55-0.69	0.57-0.71	0.40-0.73	0.65-0.78	0.54-0.68	0.50-0.64	0.64-0.82	0.88-0.99	0.83-0.95	0.61-0.87
SCD Aβ-	0.46-0.58	0.47-0.62		0.53-0.70	0.61-0.74	0.64-0.76	0.64-0.76	0.71-0.83	0.57-0.71	0.57-0.69	0.72-0.88	0.95-1	0.89-0.98	0.66-0.93
SCD Aβ+	0.51-0.66	0.47-0.64	0.53-0.70		0.49-0.66	0.52-0.67	0.40-0.75	0.63-0.78	0.53-0.67	0.44-0.59	0.61-0.81	0.89-1	0.82-0.96	0.58-0.87
MCI Aβ-	0.59-0.71	0.55-0.69	0.61-0.74	0.49-0.66		0.44-0.58	0.49-0.79	0.57-0.71	0.49-0.63	0.48-0.62	0.54-0.73	0.85-0.99	0.76-0.92	0.53-0.81
MCI Aβ+	0.62-0.72	0.57-0.71	0.64-0.76	0.52-0.67	0.44-0.58		0.49-0.79	0.57-0.70	0.49-0.63	0.51-0.63	0.53-0.72	0.86-0.98	0.75-0.92	0.52-0.81
EOAD	0.36-0.71	0.40-0.73	0.64-0.76	0.40-0.75	0.49-0.79	0.49-0.79		0.64-0.85	0.58-0.77	0.44-0.76	0.61-0.87	0.89-1	0.82-0.98	0.65-0.91
AD Dementia	0.70-0.81	0.65-0.78	0.71-0.83	0.63-0.78	0.57-0.71	0.57-0.70	0.64-0.85		0.44-0.58	0.62-0.75	0.43-0.61	0.64-0.90	0.61-0.80	0.41-0.68
FTD	0.56-0.70	0.54-0.68	0.57-0.71	0.53-0.67	0.49-0.63	0.49-0.63	0.58-0.77	0.44-0.58		0.52-0.66	0.41-0.57	0.56-0.75	0.56-0.73	0.44-0.64
PD	0.55-0.66	0.50-0.64	0.57-0.69	0.44-0.59	0.48-0.62	0.51-0.63	0.44-0.76	0.62-0.75	0.52-0.66		0.60-0.78	0.89-0.99	0.81-0.95	0.57-0.85
PDD/DLB	0.69-0.85	0.64-0.82	0.72-0.88	0.61-0.81	0.54-0.73	0.53-0.72	0.61-0.87	0.43-0.61	0.41-0.57	0.60-0.78		0.69-0.93	0.62-0.85	0.40-0.72
CBS/PSP	0.94-1	0.88-0.99	0.95-1	0.89-1	0.85-0.99	0.86-0.98	0.89-1	0.64-0.90	0.56-0.75	0.89-0.99	0.69-0.93		0.32-0.77	0.51-0.92
MSA	0.87-0.97	0.83-0.95	0.89-0.98	0.82-0.96	0.76-0.92	0.75-0.92	0.82-0.98	0.61-0.80	0.56-0.73	0.81-0.95	0.62-0.85	0.32-0.77		0.49-0.80
VaD	0.65-0.91	0.61-0.87	0.66-0.93	0.58-0.87	0.53-0.81	0.52-0.81	0.65-0.91	0.41-0.68	0.44-0.64	0.57-0.85	0.40-0.72	0.51-0.92	0.49-0.80	

Supplementary Table 7. Concentration cut-offs for plasma neurofilament light calculated in the KCL cohort

	**Concentration cut-off method**				
	**90% CI**	**95% CI**	**99% CI**	**+2SD**	**GMM**
**All ages (pg/mL)**	35.02	38.04	50.00	32.00	73.04
**>60 (pg/mL)**	37.02	46.00	79.20	35.14	39.29
**<60 (pg/mL)**	19.37	21.50	30.01	30.57	86.83

Supplementary Results 1. Demographics of the KCL and Lund cohorts

The demographic and clinical data for both KCL and Lund cohorts is displayed in Table 1 and Table 2. The KCL cohort was younger than the Lund cohort (KCL, M=63.6, SD=14.6; Lund, M=70, SD=6.3; $P<0.05$) due to the inclusion of larger groups of younger controls and certain patient groups (*e.g.* EOAD, Down syndrome and depression). Both cohorts were equally matched for sex and *APOE* ε4 carriers. In the KCL cohort, plasma NfL significantly correlated with age in the full cohort (supplementary figure 2A). However, a stronger association was observed in individuals <60 years (r=0.354, $P<0.0001$) compared to those >60 years (r=0.191, $P<0.0001$). Plasma NfL and age associations also differed by diagnostic group (supplementary table 2). In most of the groups, a significant correlation of plasma NfL existed with age although weak for FTD (r=0.175, $P=0.025$); non-significant findings (MCI Aβ+, EOAD, ALS and CBS/PSP) were also observed. Plasma NfL was significantly increased in males (M=37.0, SD=42.7) as compared to females (M=31.8, SD=34.3; $P = 0.05$); this was not a consistent finding within individual diagnostic groups, however (supplementary table 3). In the Lund cohort, similar observations were found. Plasma NfL correlated with age in the whole cohort (r=0.313, $P<0.0001$, supplementary figure 1B) and in the majority of diagnostic groups (supplementary table 2). Like the KCL cohort, weaker correlations were observed for MCI Aβ+ (ρ=0.217, $P=0.005$) and FTD (r=0.247, $P=0.005$) with non-significant correlations for EOAD, CBS/PSP and VaD. In contrast to the KCL cohort, a non-significant increase in NfL concentration in females (M=33.8pg/mL, SD=28.7) compared to males (M=31.0 pg/mL, SD=30.8) was observed in the whole population. Once more, this was not a consistent observation across diagnostic groups (supplementary table 3). There was no influence of *APOE* ε4 status on the plasma NfL levels in either cohort (Table 1 and Table 2).

Supplementary Results 2. Correlations of plasma and cerebrospinal fluid (CSF) NfL

CSF NfL data was available only for the Lund cohort. In the whole cohort, plasma NfL correlated highly with CSF NfL (r=0.640, *P*<0.0001), which remained significant after accounting for the effect of age (r=0.492, *P*<0.0001). The strongest association was observed for the parkinsonian group (r=0.700, *P*<0.0001, supplementary figure 3) with significant associations also observed for the cognitive impairment (r=0.559, *P*<0.0001) and CU groups (r=0.507, *P*<0.0001). These associations remained significant after accounting for age (parkinsonian, r=0.680, *P*<0.0001; cognitive impairment, r=0.560, *P*<0.0001; CU, r=0.250, *P*<0.0001). Within diagnostic groups, the strongest associations were observed in PDD/DLB (r=0.819, *P*<0.0001) and PD (r=0.602, *P*<0.0001) groups, whereas the MCI (r=0.561, *P*<0.0001), MSA (r=0.497, *P*< 0.01) and AD (r=0.421, *P*<0.0001) groups exhibited moderate correlations. In the unimpaired groups, a moderate correlation existed between plasma and CSF NfL (CU, r=0.497, *P*<0.0001; SCD, r=0.527, *P*<0.0001). No significant correlation as observed for VaD (r=0.201) or CBS /PSP (r=0.221).

Supplementary Results 3. Correlations of plasma NfL and disease severity.

The correlations between plasma NfL and disease severity are displayed in supplementary table 4. MMSE was used for a measure of disease severity for MCI, AD, VaD and FTD. Hoehn & Yahr scale and UPDR-III for PD and atypical parkinsonian diseases. Hamilton Rating Scale for Depression (HAM-D) and diagnostic delayed were utilized for Depression and ALS respectively. Intellectual ability and CAMDEX-DS were investigated for Down syndrome. In both the KCL and Lund cohorts, no significant association was found between plasma NfL and MMSE in MCI and AD groups. This was also observed for PDD/DLB, FTD, EOAD, VaD. Increased plasma NfL was associated with both UPDRS-III and Hoehn & Yehr in PD patients (P <0.005, for both analysis) but not PDD/DLB and PSP/CBS. In MSA patients, increased plasma NfL was significantly associated with UPDRS-III (P <0.001) but not Hoehn & Yehr. For the ALS patients, a measure of disease severity was calculated using diagnostic delay, which corresponds to rate of disease progression. In the ALS sample, the diagnostic delay was 7.79 months, IQR (2.12-.11.97). Median survival was 26.79 months (95% CI 21.31-49.75). In all cases, patients had El Escorial ALS and therefore clinical involvement of both upper (1st) and lower (2nd) motor neurons, except one patient who had a UMN-predominant phenotype. No association between plasma NfL and diagnostic delay was observed. In the Down syndrome group, plasma NfL did not associate with intellectual disability (mild, moderate, severe) but significantly correlated with CAMDEX-DS (r=0.487, P = 0.003).

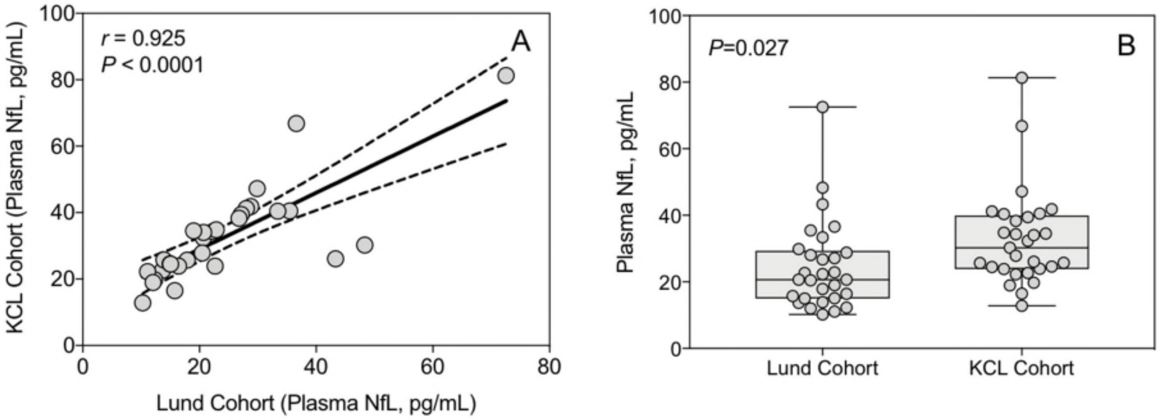

Supplementary Figure 1. Comparison of quality control (QC, n=30) samples between KCL and Lund cohorts**.** QC samples were analyzed in the Lund cohort at then at random in the KCL analysis. (A) demonstrates the correlation between identical QC samples (P=3.2x10^{-6}) and (B) show the differences in absolute quantification. The data (A) are shown as Spearman correlation coefficients. The boxes show interquartile range, the horizontal lines are medians and the whiskers were plotted for minimum and maximum data points. An unpaired t test determined the significance. Source data are provided as a Source Data file

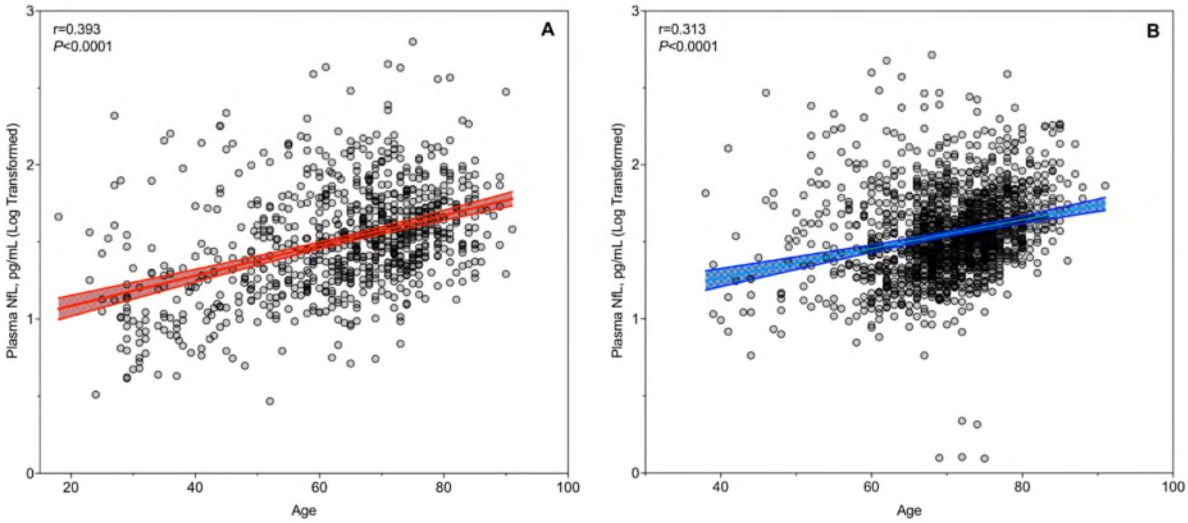

Supplementary Figure 2. The correlations of plasma NfL and age in the KCL (A, n=805, P= P=1.2x10^{-8}) and Lund (B, n=1464, P=6.3x10^{-6}) cohorts. The data are shown as Spearman correlation coefficients. The error bands represent the 95% confidence interval of the line of best fit.

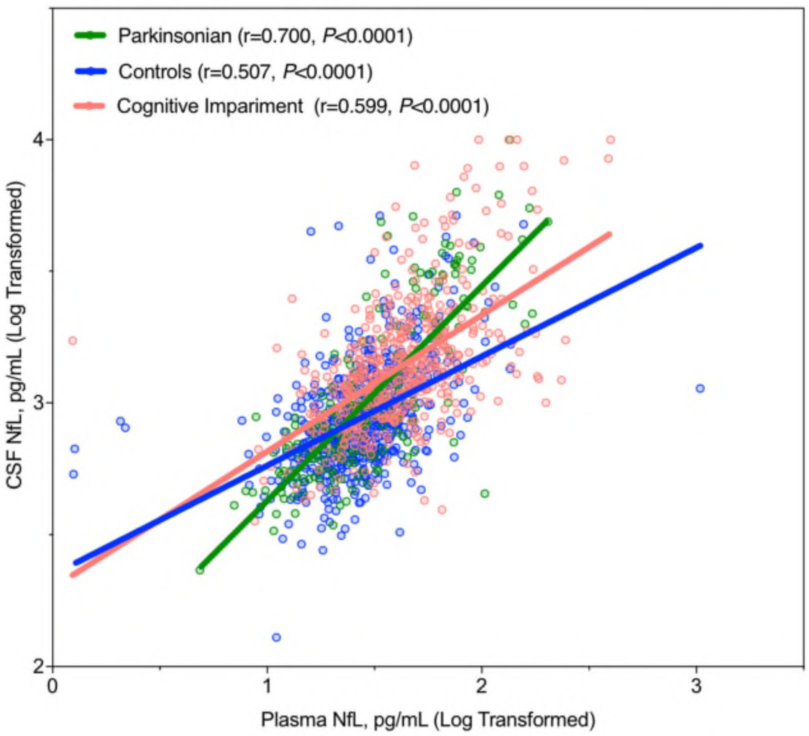

Supplementary Figure 3. Correlations of plasma NfL and CSF NfL in the Lund cohort unadjusted for age. These associations remained significant after accounting for effect of age (parkinsonian, r=0.680, P=3.3x10^{-8}; cognitive impairment, r=0.560, P=2.2x10^{-7}; CU, r=0.250, P=5.0x10^{-7}). The data are shown as Spearman correlation coefficients.

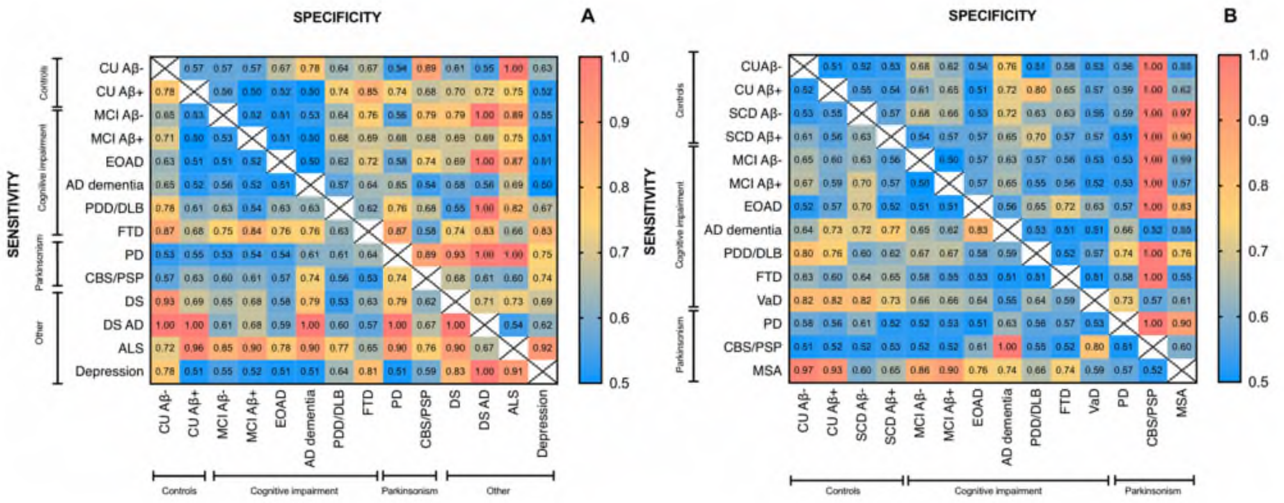

Supplementary Figure 4. The sensitivity and specificities of plasma NfL to differentiate healthy controls, neurodegenerative and neurological disorders in the KCL (A) and Lund (B) cohorts

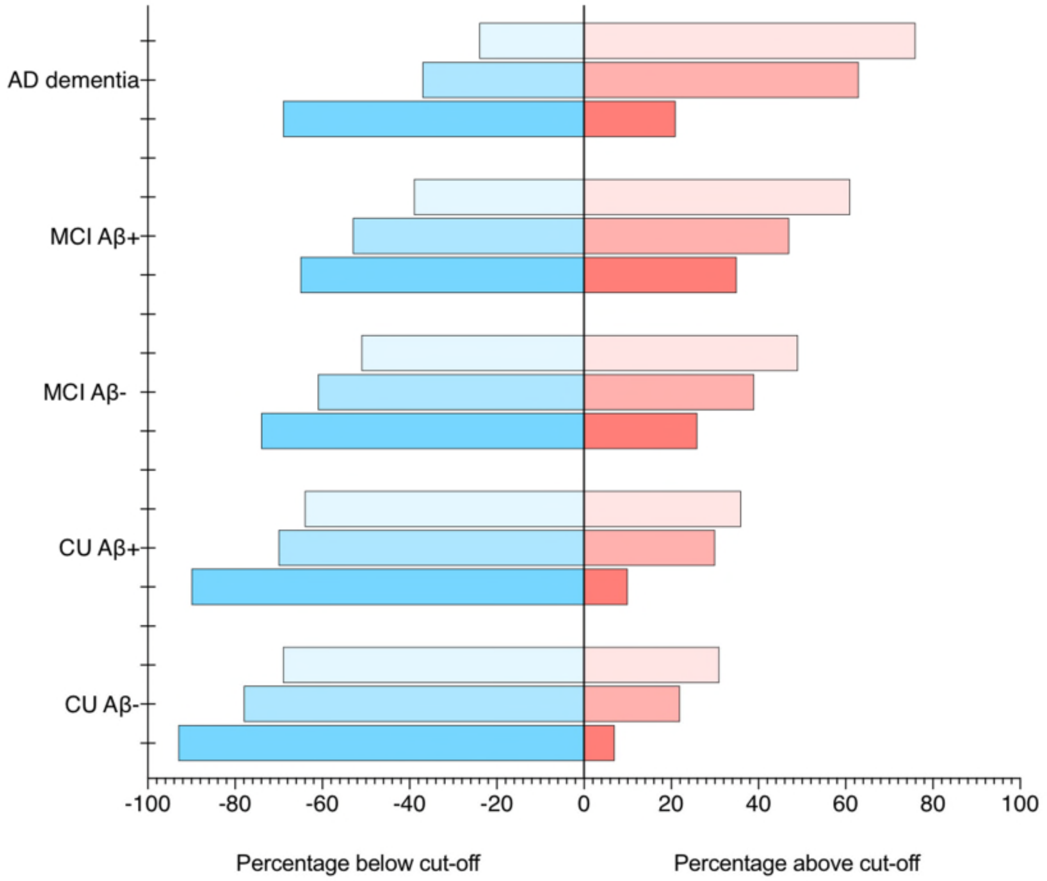

Supplementary Figure 5. The performance of plasma neurofilament light (NfL) concentration cut-offs to identify neurodegenerative disorders in ADNI. Source data are provided as a Source Data file

Supplementary References

1 Ferrucci, L. The Baltimore Longitudinal Study of Aging (BLSA): a 50-year-long journey and plans for the future. *J Gerontol A Biol Sci Med Sci* **63**, 1416-1419, doi:10.1093/gerona/63.12.1416 (2008).

2 Pilotto, A. *et al.* Extrastriatal dopaminergic and serotonergic pathways in Parkinson's disease and in dementia with Lewy bodies: a (123)I-FP-CIT SPECT study. *Eur J Nucl Med Mol Imaging* **46**, 1642-1651, doi:10.1007/s00259-019-04324-5 (2019).

3 Postuma, R. B. *et al.* MDS clinical diagnostic criteria for Parkinson's disease. *Mov Disord* **30**, 1591-1601, doi:10.1002/mds.26424 (2015).

4 Emre, M. *et al.* Clinical diagnostic criteria for dementia associated with Parkinson's disease. *Mov Disord* **22**, 1689-1707; quiz 1837, doi:10.1002/mds.21507 (2007).

5 McKeith, I. G. *et al.* Diagnosis and management of dementia with Lewy bodies: third report of the DLB Consortium. *Neurology* **65**, 1863-1872, doi:10.1212/01.wnl.0000187889.17253.b1 (2005).

6 Hughes, A. J., Daniel, S. E., Kilford, L. & Lees, A. J. Accuracy of clinical diagnosis of idiopathic Parkinson's disease: a clinico-pathological study of 100 cases. *J Neurol Neurosurg Psychiatry* **55**, 181-184, doi:10.1136/jnnp.55.3.181 (1992).

7 Lovestone, S. *et al.* AddNeuroMed--the European collaboration for the discovery of novel biomarkers for Alzheimer's disease. *Ann N Y Acad Sci* **1180**, 36-46, doi:10.1111/j.1749-6632.2009.05064.x (2009).

8 McKhann, G. *et al.* Clinical diagnosis of Alzheimer's disease: report of the NINCDS-ADRDA Work Group under the auspices of Department of Health and Human Services Task Force on Alzheimer's Disease. *Neurology* **34**, 939-944, doi:10.1212/wnl.34.7.939 (1984).

9 FernAndez-Eulate, G. *et al.* A comprehensive serum lipidome profiling of amyotrophic lateral sclerosis. *Amyotroph Lateral Scler Frontotemporal Degener* **21**, 252-262, doi:10.1080/21678421.2020.1730904 (2020).

10 Ludolph, A. *et al.* A revision of the El Escorial criteria - 2015. *Amyotroph Lateral Scler Frontotemporal Degener* **16**, 291-292, doi:10.3109/21678421.2015.1049183 (2015).

11 Roche, J. C. *et al.* A proposed staging system for amyotrophic lateral sclerosis. *Brain* **135**, 847-852, doi:10.1093/brain/awr351 (2012).

12 Westwood, S. *et al.* Plasma Protein Biomarkers for the Prediction of CSF Amyloid and Tau and [(18)F]-Flutemetamol PET Scan Result. *Front Aging Neurosci* **10**, 409, doi:10.3389/fnagi.2018.00409 (2018).

13 Alcolea, D. *et al.* Relationship between beta-Secretase, inflammation and core cerebrospinal fluid biomarkers for Alzheimer's disease. *J Alzheimers Dis* **42**, 157-167, doi:10.3233/JAD-140240 (2014).

14 Galimberti, D. *et al.* Circulating miRNAs as potential biomarkers in Alzheimer's disease. *J Alzheimers Dis* **42**, 1261-1267, doi:10.3233/JAD-140756 (2014).

15 Majbour, N. K. *et al.* Increased levels of CSF total but not oligomeric or phosphorylated forms of alpha-synuclein in patients diagnosed with probable Alzheimer's disease. *Sci Rep* **7**, 40263, doi:10.1038/srep40263 (2017).

16 Petersen, R. C. Mild cognitive impairment as a diagnostic entity. *J Intern Med* **256**, 183-194, doi:10.1111/j.1365-2796.2004.01388.x (2004).

17 Baillon, S. *et al.* Prevalence and Severity of Neuropsychiatric Symptoms in Early-Versus Late-Onset Alzheimer's Disease. *Am J Alzheimers Dis Other Demen* **34**, 433-438, doi:10.1177/1533317519841191 (2019).

18 Velayudhan, L. *et al.* Smell identification function in early-onset Alzheimer's disease and mild cognitive impairment. *Int Psychogeriatr*, 1-6, doi:10.1017/S1041610218001503 (2018).
19 Baek, J. H. *et al.* GRP78 Level Is Altered in the Brain, but Not in Plasma or Cerebrospinal Fluid in Parkinson's Disease Patients. *Front Neurosci* **13**, 697, doi:10.3389/fnins.2019.00697 (2019).
20 Gelb, D. J., Oliver, E. & Gilman, S. Diagnostic criteria for Parkinson disease. *Arch Neurol* **56**, 33-39, doi:10.1001/archneur.56.1.33 (1999).
21 Startin, C. M. *et al.* The LonDownS adult cognitive assessment to study cognitive abilities and decline in Down syndrome. *Wellcome Open Res* **1**, 11, doi:10.12688/wellcomeopenres.9961.1 (2016).
22 Sheehan, R. *et al.* Dementia diagnostic criteria in Down syndrome. *Int J Geriatr Psychiatry* **30**, 857-863, doi:10.1002/gps.4228 (2015).
23 Therriault, J. *et al.* Association of Apolipoprotein E epsilon4 With Medial Temporal Tau Independent of Amyloid-beta. *JAMA Neurol* **77**, 470-479, doi:10.1001/jamaneurol.2019.4421 (2020).
24 Snowden, J. S. *et al.* The clinical diagnosis of early-onset dementias: diagnostic accuracy and clinicopathological relationships. *Brain* **134**, 2478-2492, doi:10.1093/brain/awr189 (2011).
25 McKhann, G. M. *et al.* The diagnosis of dementia due to Alzheimer's disease: recommendations from the National Institute on Aging-Alzheimer's Association workgroups on diagnostic guidelines for Alzheimer's disease. *Alzheimers Dement* **7**, 263-269, doi:10.1016/j.jalz.2011.03.005 (2011).
26 Neary, D., Snowden, J. & Mann, D. Frontotemporal dementia. *Lancet Neurol* **4**, 771-780, doi:10.1016/S1474-4422(05)70223-4 (2005).
27 Nikkheslat, N. *et al.* Childhood trauma, HPA axis activity and antidepressant response in patients with depression. *Brain Behav Immun* **87**, 229-237, doi:10.1016/j.bbi.2019.11.024 (2020).
28 Rabinovici, G. D. *et al.* Amyloid vs FDG-PET in the differential diagnosis of AD and FTLD. *Neurology* **77**, 2034-2042, doi:10.1212/WNL.0b013e31823b9c5e (2011).
29 Palmqvist, S. *et al.* Performance of Fully Automated Plasma Assays as Screening Tests for Alzheimer Disease-Related beta-Amyloid Status. *JAMA Neurol*, doi:10.1001/jamaneurol.2019.1632 (2019).
30 Rascovsky, K. *et al.* Sensitivity of revised diagnostic criteria for the behavioural variant of frontotemporal dementia. *Brain* **134**, 2456-2477, doi:10.1093/brain/awr179 (2011).
31 Gilman, S. *et al.* Consensus statement on the diagnosis of multiple system atrophy. *J Neurol Sci* **163**, 94-98, doi:10.1016/s0022-510x(98)00304-9 (1999).
32 Litvan, I. *et al.* Clinical research criteria for the diagnosis of progressive supranuclear palsy (Steele-Richardson-Olszewski syndrome): report of the NINDS-SPSP international workshop. *Neurology* **47**, 1-9, doi:10.1212/wnl.47.1.1 (1996).
33 Litvan, I. *et al.* Validity and reliability of the preliminary NINDS neuropathologic criteria for progressive supranuclear palsy and related disorders. *J Neuropathol Exp Neurol* **55**, 97-105, doi:10.1097/00005072-199601000-00010 (1996).
34 Santillo, A. F. *et al.* Diffusion tensor tractography versus volumetric imaging in the diagnosis of behavioral variant frontotemporal dementia. *PLoS One* **8**, e66932, doi:10.1371/journal.pone.0066932 (2013).
35 Meeter, L. H. H. *et al.* Clinical value of neurofilament and phospho-tau/tau ratio in the frontotemporal dementia spectrum. *Neurology* **90**, e1231-e1239, doi:10.1212/WNL.0000000000005261 (2018).
