# Supplementary material for: A multicentre validation study of the diagnostic value of plasma neurofilament light
Source: Nat Commun. 2021 Jun 7;12:3400. doi: 10.1038/s41467-021-23620-z (PMC8185001; doi:10.1038/s41467-021-23620-z)
Supplement: Supplementary file 2 — Reporting summary [file 41467_2021_23620_MOESM2_ESM.pdf]

nature research

Corresponding author(s): Dr. Nicholas Ashton
Professor Oskar Hansson

Last updated by author(s): 28/3/20

Reporting Summary

Nature Research wishes to improve the reproducibility of the work that we publish. This form provides structure for consistency and transparency in reporting. For further information on Nature Research policies, see our Editorial Policies and the Editorial Policy Checklist.

Statistics

For all statistical analyses, confirm that the following items are present in the figure legend, table legend, main text, or Methods section.

n/a	Confirmed	
☐	☒	The exact sample size (n) for each experimental group/condition, given as a discrete number and unit of measurement
☒	☐	A statement on whether measurements were taken from distinct samples or whether the same sample was measured repeatedly
☐	☒	The statistical test(s) used AND whether they are one- or two-sided<br>*Only common tests should be described solely by name; describe more complex techniques in the Methods section.*
☐	☒	A description of all covariates tested
☐	☒	A description of any assumptions or corrections, such as tests of normality and adjustment for multiple comparisons
☐	☒	A full description of the statistical parameters including central tendency (e.g. means) or other basic estimates (e.g. regression coefficient) AND variation (e.g. standard deviation) or associated estimates of uncertainty (e.g. confidence intervals)
☐	☒	For null hypothesis testing, the test statistic (e.g. F, t, r) with confidence intervals, effect sizes, degrees of freedom and P value noted<br>*Give P values as exact values whenever suitable.*
☒	☐	For Bayesian analysis, information on the choice of priors and Markov chain Monte Carlo settings
☒	☐	For hierarchical and complex designs, identification of the appropriate level for tests and full reporting of outcomes
☒	☐	Estimates of effect sizes (e.g. Cohen's d, Pearson's r), indicating how they were calculated

Our web collection on statistics for biologists contains articles on many of the points above.

Software and code

Policy information about availability of computer code

Data collection	*Provide a description of all commercial, open source and custom code used to collect the data in this study, specifying the version used OR state that no software was used.*
Data analysis	SPSS version 24 (IBM, Armonk, NY, US), R studio and R version 3.4.3 (packages pROC and AUC); Prism 8.

For manuscripts utilizing custom algorithms or software that are central to the research but not yet described in published literature, software must be made available to editors and reviewers. We strongly encourage code deposition in a community repository (e.g. GitHub). See the Nature Research guidelines for submitting code & software for further information.

Data

Policy information about availability of data

All manuscripts must include a data availability statement. This statement should provide the following information, where applicable:

- Accession codes, unique identifiers, or web links for publicly available datasets
- A list of figures that have associated raw data
- A description of any restrictions on data availability

Anonymized data will be shared by request from any qualified investigator for the sole purpose of replicating procedures and results presented in the article and as long as data transfer is in agreement with EU legislation on the general data protection regulation.

Databases used:
Alzheimer's disease Neuroimaging Initiative (ADNI) database

Field-specific reporting

Please select the one below that is the best fit for your research. If you are not sure, read the appropriate sections before making your selection.

- [x] Life sciences
- [ ] Behavioural & social sciences
- [ ] Ecological, evolutionary & environmental sciences

For a reference copy of the document with all sections, see nature.com/documents/nr-reporting-summary-flat.pdf

Life sciences study design

All studies must disclose on these points even when the disclosure is negative.

Sample size	The study included two prospective cohorts with large sample size (n=805 and n=1464). All three cohorts were convenience cohorts and all available plasma samples were analyzed in this study.
Data exclusions	No outliers were excluded from the study based on NfL values. Controls, MCI and AD cases without CSF or PET biomarker confirmation were excluded, as AD pathology could not been confirmed which was a predetermined criteria of the study.
Replication	The two cohorts were designed to be as similar as possible. However, some disorders were not overlapping but deemed complimentary to the study.
Randomization	In these cohorts (observational studies) no allocation into experimental groups were performed, therefore randomization is not relevant to this study.
Blinding	All plasma NFL analyses were performed by individuals who were blinded to the clinical data and group allocation. Investigators were aware if a sample came from the KCL cohort or Lund Cohort.

Reporting for specific materials, systems and methods

We require information from authors about some types of materials, experimental systems and methods used in many studies. Here, indicate whether each material, system or method listed is relevant to your study. If you are not sure if a list item applies to your research, read the appropriate section before selecting a response.

Materials & experimental systems

n/a	Involved in the study	
x		Antibodies
x		Eukaryotic cell lines
x		Palaeontology and archaeology
x		Animals and other organisms
	x	Human research participants
	x	Clinical data
x		Dual use research of concern

Methods

n/a	Involved in the study	
x		ChIP-seq
x		Flow cytometry
x		MRI-based neuroimaging

Human research participants

Policy information about studies involving human research participants

Population characteristics	Detailed information is given in table 1 and table 2. In short, we present results for analyses from two different cohorts. Cohort 1 and cohort 2 had similar study designs, but cohort 1 was multicentric in nature. Cohort 1 included 805 participants; CU individuals (n=158), mild cognitive impairment (MCI, n=86), early-onset Alzheimer's disease (EOAD <65 years, n=59), AD dementia (n=102), FTD (n=54), PD (n=140), Parkinson's disease dementia and dementia with Lewy bodies (PDD/DLB, n=59), corticalbasal syndrome and progressive supranuclear palsy (CBS/PSP, n=19), Down Syndrome (DS, n=41; 12 with dementia), amyotrophic lateral sclerosis (ALS, n=50), and depression (HAM-D>13, n=37). Cohort 2 included CU (n=376), subjective cognitive decline (SCD, n=209) and seven diagnostic groups in common with the KCL cohort (MCI, n=280; EOAD <65 years, n=23; AD dementia, n=134; FTD, n=150; PD, n=171; PDD/DLB, n=46; CBS/PSP, n=24). In addition, the Lund cohort included patients with multiple system atrophy (MSA, n=29) and vascular dementia (VaD, n=22). A total of 870 individuals (CU, n=290; MCI, n=442; AD dementia, n=138) from the ADNI dataset were also included.
Recruitment	This project was done as part of the prospective Swedish BioFINDER study, Erasmus Medical Centre, Rotterdam, The Netherlands and Lund Prospective Frontotemporal Dementia Study (LUPROFS). The KCL cohort represents a multicenter collection of participants from prospective and longitudinal studies detailed in supplementary Table 1. These sample were collated and measured at the Maurice Wohl Clinical Neuroscience institute, King's College London. Recruitment of patients with cognitive symptoms or neurological diseases was performed at specialist clinics or in Alzheimer's disease research cohorts (e.g., ADNI), thus the results for the patients may therefore be bias for a specialized setting or overrepresented a clinic group compared to the general population.
Ethics oversight	The study was approved by the Regional Ethics Committee for each cohort and material transfer agreements were arranged for the shipment to measure at KCL.in Lund, Sweden. All participants gave their informed consent to participate in the study.

Note that full information on the approval of the study protocol must also be provided in the manuscript.

Clinical data

Policy information about clinical studies

All manuscripts should comply with the ICMJEguidelines for publication of clinical research and a completedCONSORT checklist must be included with all submissions.

Clinical trial registration	NCT01208675 (BioFINDER)
Study protocol	Observational
Data collection	Full details were provided at https://clinicaltrials.gov/ct2/show/NCT01208675?term=biofinder&rank=2. Cohort 2 (Lund) are part of the prospective Swedish BioFINDER study with participants recruited at Skåne University Hospital and the Hospital of Ängelholm, Sweden. Enrolled between November 2014 and January 2018 and cohort 2 included non-demented participants enrolled between January 2010 and December 2014. Data was collected between January 2010 and May 2019.
Outcomes	Primary Outcome: To compare the time to conversion to clinically probable AD in MCI subjects or healthy elderly subjects with normal and abnormal biomarkers (CSF, blood, MRI, PET) [Time Frame: Time zero equals the baseline visit. All subjects will subsequently attend follow-up visits every year for approximately 4-6 years after baseline.]<br><br>Secondary Outcome:<br>Rate of cognitive decline as measured by various cognitive tests, Activities of Daily Living (FAQ) and Global Deterioration Scale. All subjects will subsequently attend follow-up visits every year for approximately 10 years after baseline. Group differences for imaging and wet biomarker measurements. Rate of volume change of structural MRI measures and amyloid PET. Rates of change on each specified biochemical biomarker. Correlations between biomarkers and biomarker change.<br><br>Subgroups analyses: Abnormal CSF biomarkers, positive amyloid imaging, APOE genotype
